# Substrate Scope for Human Histone Lysine Acetyltransferase KAT8

**DOI:** 10.3390/ijms22020846

**Published:** 2021-01-15

**Authors:** Giordano Proietti, Yali Wang, Chiara Punzo, Jasmin Mecinović

**Affiliations:** 1 Department of Physics, Chemistry and Pharmacy, University of Southern Denmark, Campusvej 55, 5230 Odense, Denmark; proietti@sdu.dk (G.P.); chiarapunzo.94@gmail.com (C.P.); 2 Institute for Molecules and Materials, Radboud University, Heyendaalseweg 135, 6525 AJ Nijmegen, The Netherlands; wangzhang4083@jlu.edu.cn; 3 Department of Blood Transfusion, Jilin University, 126 Xiantai Street, Changchun 130033, China

**Keywords:** acetylation, epigenetics, histone, lysine, posttranslational modifications

## Abstract

Biomedically important histone lysine acetyltransferase KAT8 catalyses the acetyl coenzyme A-dependent acetylation of lysine on histone and other proteins. Here, we explore the ability of human KAT8 to catalyse the acetylation of histone H4 peptides possessing lysine and its analogues at position 16 (H4K16). Our synthetic and enzymatic studies on chemically and structurally diverse lysine mimics demonstrate that KAT8 also has a capacity to acetylate selected lysine analogues that possess subtle changes on the side chain and main chain. Overall, this work highlights that KAT8 has a broader substrate scope beyond natural lysine, and contributes to the design of new chemical probes targeting KAT8 and other members of the histone lysine acetyltransferase (KAT) family.

## 1. Introduction

**Figure 1:**
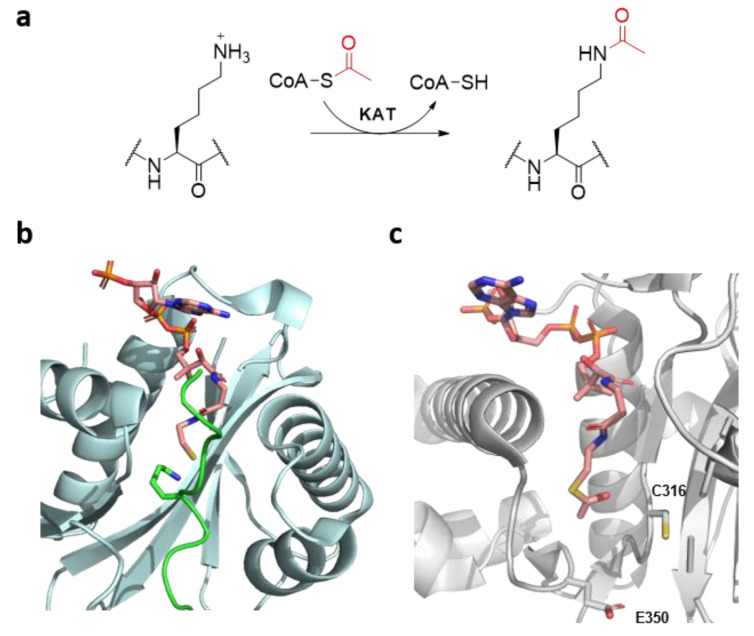
(**a**) Histone lysine acetylation by histone lysine acetyltransferases (KATs). (**b**) View of the crystal structure of the tKAT2A-H3K14-CoASH complex (tKAT2A: cyan; CoASH: pink; H3K14: green) (PDB: 1QSN). (**c**) View of the crystal structure of the KAT8-AcCoA complex (KAT8: gray; AcCoA: pink) (PDB: 2GIV), highlighting the two catalytically relevant residues C316 and E350.

In eukaryotes, the installation of posttranslational modifications (PTMs) on histone proteins is a fundamental biomolecular process that regulates important cellular pathways, such as gene expression, DNA repair and apoptosis [1,2]. Among the plethora of histone chemical marks identified to date, lysine acetylation is one of the most widespread [3]. Histone lysine acetylation is catalysed by histone lysine acetyltransferases (KATs), which employ the naturally abundant acetyl coenzyme A (AcCoA) as a co-substrate. KATs catalyse the transfer of an acetyl moiety from AcCoA onto the N^ε^-amino group of lysine residues on histone tails and non-histone proteins (Figure 1a) [4]. Histone lysine deacetylases (KDACs) catalyse the removal of the acetyl group from acetylated lysine residues, guaranteeing a fine regulation of histone acetylation levels [5], while acetylated lysine residues are recognised by bromodomains, activating a downstream cascade of transcriptional factors [4,6]. Histone acetylation is generally linked with enhanced genes transcription by the production of euchromatin, a more loose form of chromatin, as a result of the removal of the lysine’s positive charge [7].

A multitude of KATs are found in the nucleus, and can be divided in three big subfamilies by their sequence homology (GNAT, p300/CBP and MYST families), possessing different substrate specificities, catalytic mechanisms and multi-subunit binding partners [8]. Members of the GNAT and p300/CBP families acetylate lysine through a “direct” mechanism upon the formation of the ternary complex KAT-Histone-AcCoA [8]. Members of the MYST family, however, display a characteristic ping-pong mechanism, exploiting a conserved cysteine residue, which forms a highly reactive S-acetylcysteine thioester in the active site as a key kinetic intermediate before the acetyl transfer onto the N^ε^-amino group of lysine [8]. Nevertheless, in all KATs, the deprotonation of the lysine N^ε^-amino group is achieved by highly conserved glutamic or aspartic acid in the active site [8]. In addition to KATs’ renowned ability of transferring bulkier acyl groups from their respective acyl-CoA co-substrates [9], recent investigations shed light on the ability of KATs to catalyse the acetylation of lysine analogues with a longer chain length [10], and the acylation of γ-thialysine, a simple lysine analogue site-specifically introduced on histone tails [11]. Despite the recent striking progress achieved in the field of epigenetic drug discovery, due to the Food and Drug Administration (FDA) approval of several KDAC inhibitors for the treatment of different cancers and numerous clinical candidates targeting bromodomains, we are currently lacking potent and specific inhibitors or modulators of KAT activity [12,13]. One of the major drawbacks contributing to the limited chemical and biochemical understanding of KATs is the lack of biostructural information, due to the well-known challenges in obtaining the crystal structure of a ternary complex KAT-Histone-AcCoA/CoASH (Figure 1b) and the yet unexplored biocatalytic potential of KATs [14,15,16]. 

KAT8 (also known as MOF or MYST1), one of the less characterised members of the MYST family, is an important KAT that catalyses the acetylation of lysine 16 on histone 4 (H4K16), a critical histone mark linked with both DNA damage response and enhanced transcription [17,18], as well as biomedically relevant non-histone proteins, including p53 [8]. As a member of the MYST family, E350 and C316 are the two essential residues for the enzyme’s ping-pong catalytic mechanism (Figure 1c) [8]. Here, we investigate the substrate scope for human KAT8 by exploring the lysine chemical space to obtain information on the substrate specificity and biocatalytic potential.

## 2. Results and Discussion

### 2.1. Selection and Synthesis of KAT8 Peptide Substrates

**Figure 2:**
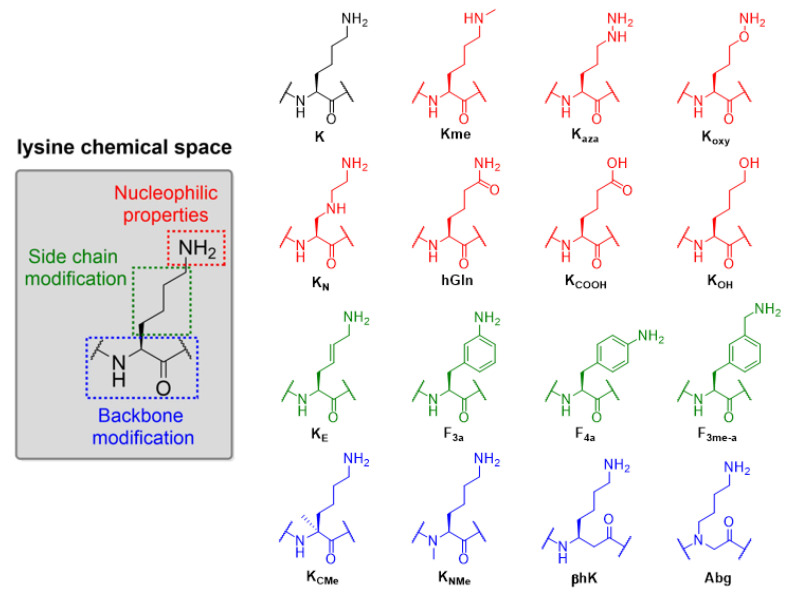
Exploring the substrate specificity for human KAT8 acetyltransferase, rationale and the panel of selected lysine analogues.

To explore the substrate scope of KAT8 catalysis, we selected a panel of 15 structurally and chemically different lysine analogues that differ in: (i) the nucleophilic nature of the terminal N^ε^-amino moiety; (ii) lysine side chain, and (iii) lysine main chain (Figure 2). Recombinant human KAT8 (residues 125–458) was expressed in *E. coli* and purified by Ni-affinity and size-exclusion chromatography. Fmoc-protected lysine analogues, synthetic and commercially available, were introduced into the KAT8 preferential site of acetylation (H4K16, residues 13–27, sequence: GGAK_16_RHRKVLRDNIQ) by solid-phase peptide synthesis (SPPS) employing Fmoc/Boc chemistry (Scheme S1). The synthetic histone peptides were purified by preparative RP-HPLC and their purity was assessed by both MALDI-TOF MS and analytical RP-HPLC (Figures S1–S16).

### 2.2. KAT8 Enzymatic Assays

**Figure 3:**
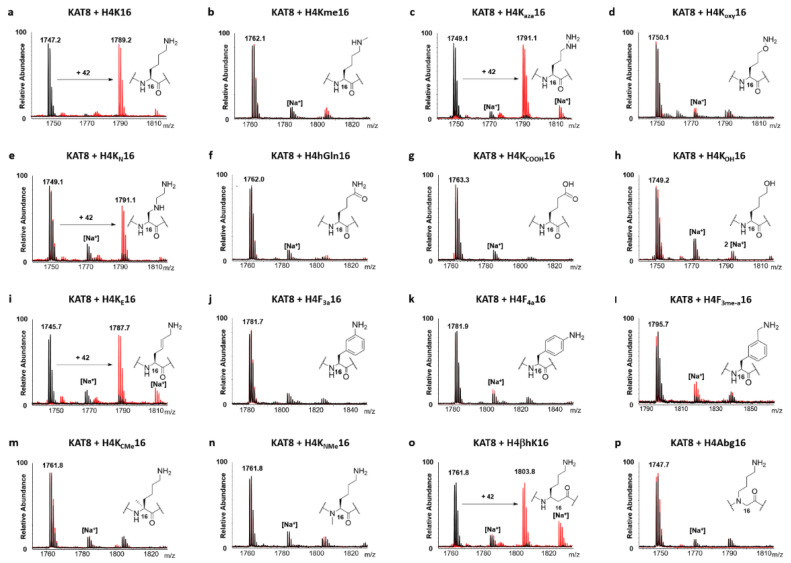
MALDI-TOF MS data showing the KAT8 (2 μM)-catalysed acetylation of H4K*16 histone peptides (100 μM) in the presence of AcCoA co-substrate (300 μM) (**a**–**p**); Overlaid MS spectra of KAT8-catalysed reactions (red) and no enzyme control reactions (black) that were quenched after being incubated for 3 h at 37 °C.

The biocatalytic potential of KAT8 was assessed through a MALDI-TOF MS based enzymatic assay [10,11]. Human KAT8 was incubated in the presence of H4K*16 peptides and AcCoA under “standard conditions” (2 µM KAT8, 100 µM histone peptide, 300 µM AcCoA; assay buffer: 50 mM Hepes, 0.1 mM EDTA, 1 mM DTT, pH = 8.0) and the production of acetylated peptides was monitored at different time points. Controls in the absence of KAT8 were carried out in parallel to demonstrate the enzymatic nature of the acetyl transfer reactions. KAT8-catalysed acetylation of H4K16 was quantitative after 1 h, in line with previous findings (Figure 3a and Figure S17) [10,11]. We examined the substrate specificity of the natural lysine analogue Kme, observing that it did not undergo KAT8-catalysed acetylation (Figure 3b), even after prolonged incubation and higher enzyme concentration (6 h; 10 µM KAT8) (Figure S18a), indicating that sterics on N^ε^ play an important role in efficient KAT8 catalysis. This is an interesting observation, given the well-documented capacity of some histone lysine methyltransferases (KMTs) that introduce higher methylation states (i.e., Kme2 and Kme3) on histone tails starting from Kme as a substrate [19]. Analysis of lysine’s nucleophilic properties revealed that the introduction of O or NH adjacent to lysine N^ε^-amino moiety allows the fine-tuning of KAT8’s catalytic properties. H4K_aza_16 appeared to be an excellent substrate for KAT8, with the production of 93% of acetylated product after 1 h and reaching completion within 3 h (Figure 3c and Figure S17). Strikingly, H4K_oxy_16 was not a substrate for KAT8 (Figure 3d), with no acetylation detected even after prolonged incubation and higher enzyme concentration (6 h; 10 µM KAT8) (Figure S18b). A possible explanation may lie on the electronic repulsion between the oxygen atom at the ε position of K_oxy_ and the negatively charged E305, which is located only 3.7 Å away from E350, the residue involved in the deprotonation of lysine’s N^ε^-amino group (Figure S19). These results demonstrate a difference in substrate specificity between KAT8 and KMTs, where both K_aza_- and K_oxy_-containing histone peptides underwent efficient methylation [20]. Shifting the electron withdrawing nitrogen atom to the position γ (K_N_) [21] resulted in a poorer KAT8 substrate, with the production of only 45% of the H4K_N_16ac peptide after 3 h under standard conditions (Figure 3e and Figure S17), and quantitative in the presence of a higher concentration of KAT8 (10 µM) and extended incubation time (6 h) (Figure S20). This result can be considered more comprehensively with the latest findings, where γ-thialysine was found to be an excellent substrate for KAT8 catalysis [11]. Under this light, the KAT8 substrate specificity for lysine and its analogues bearing a heteroatom at position γ can be updated to S ≥ C > N, with nitrogen being the most electron withdrawing group of the series, and therefore yielding the poorest N^ε^ nucleophile. Next, we examined histone peptides containing the resonance-stabilised nucleophiles hGln and K_COOH_, and found that they did not undergo KAT8-catalysed acetylation within the limit of detection (Figure 3f,g), even after prolonged incubation and additional enzyme (6 h; 10 µM KAT8) (Figure S18c,d). Substituting the nucleophilic N^ε^-amino to alcohol resulted in the loss of acetylation catalysis (Figure 3h), indicating that esters cannot be produced by KAT8, also under optimised conditions (10 µM KAT8, 6 h, Figure S18e) (the production of CoASH product was also not detected by MALDI-TOF). From a mechanistic point of view, KAT8′s inability to perform an O-acetyltransferase reaction may be linked with the nature of the catalytic base in the active site. In fact, despite the highly conserved glutamic acid (E350) in KAT8, the presence of His has been suggested to be a stringent structural prerequisite for achieving alcohol deprotonation and subsequent acetyl transfer in O-acyltransferases [22]. Moreover, we believe that this finding cannot be attributed to the induced degradation of a potential ester product during laser desorption ionization in MALDI-TOF analysis, in line with previous studies [23].

Subsequently, we investigated the role of the geometrically constrained and steric lysine analogues for KAT8 catalysis. The KAT8-catalysed reaction of H4K_E_16 produced 64% of acetylated peptide after 1 h, and reached completion within 3 h (Figure 3i and Figure S17). On the other hand, none of the sterically demanding lysine analogues, F_4a_, F_3a_ and F_3me-a_, underwent acetylation by KAT8 within the limit of detection under standard conditions (Figure 3j–l), or optimised conditions (6 h, 10 µM KAT8) (Figure S18f–h). Interestingly, the result seems to not be solely dependent on the decreased nucleophilicity of the aniline-containing analogues (F_4a_, F_3a_), due to the fact that the alkyl aromatic amine-containing peptide (F_3me-a_) also did not undergo KAT8 acetylation, even though it is structurally related to homolysine (hK), an example of a good KAT8 substrate [10]. The results are in line with recent findings on human KMTs, showing a lack of methylation of bulkier lysine analogues [24]. 

Evaluation of the role of the lysine main chain on efficient KAT8 catalysis revealed that only H4βhK16 underwent a KAT8-catalysed acetylation reaction (55% in 1 h and 80% in 3 h) (Figure 3o and Figure S17). All other analogues—K_CMe_, K_NMe_ and Abg—were not accepted as KAT8 substrates within the detection limits (Figure 3m,n,p). Prolonged incubation (6 h) with higher concentrations of enzyme (10 µM KAT8) yielded quantitative acetylation of H4βhK16 (Figure S21), 16% of acetylated H4Abg16, traces (< 5%) of H4K_NMe_16, but no acetylated H4K_CMe_16 (Figure S18i–k). These findings demonstrate the importance of surface interactions, including H-bonding, between the KAT8 catalytic site and the histone tail backbone, which appear to be essential in both interactions of tKAT2a-H3K14 peptide and KAT1-H4K12 peptide (Figure 1b, Figures S22 and S23) [25,26]. These results are in line with previous findings on KMTs, indicating that the integrity of the lysine’s backbone is a common structural prerequisite for efficient KMT and KAT catalysis [27].

### 2.3. Kinetics Analysis

**Figure 4:**
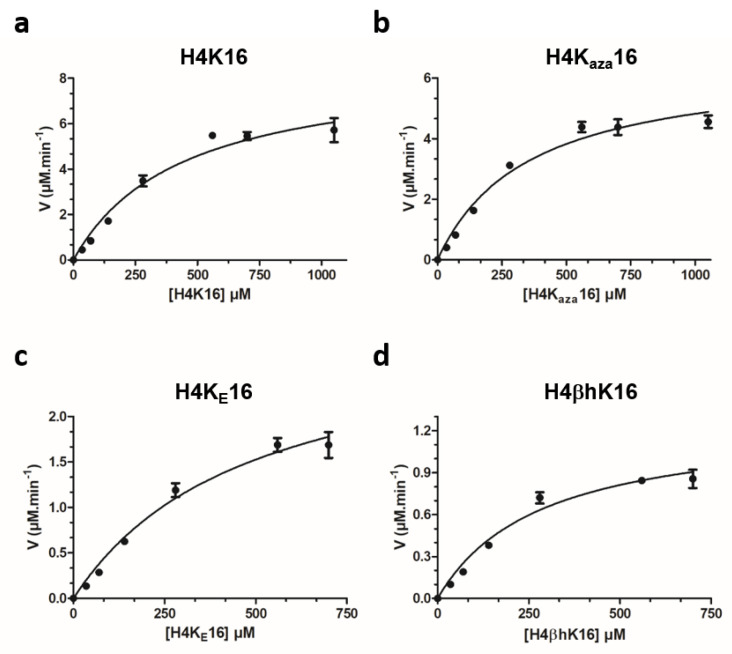
Michaelis–Menten plots of the KAT8-catalysed acetylation of: (**a**) H4K16; (**b**) H4K_aza_16; (**c**) H4K_E_16, and (**d**) H4βhK16.

Next, we investigated the catalytic efficiencies for the histone peptides that were observed as KAT8 substrates. Kinetics analyses were carried out under steady-state conditions by incubating saturating concentrations of AcCoA against different concentrations of histone peptides. By the direct comparison of relative catalytic efficiencies ([k_cat_/K_M_]H4K*16/[k_cat_/K_M_]H4K16), all the peptides bearing unnatural lysine analogues were found to be either comparable or less efficient KAT8 substrates than H4K16. The effect was found to be generally driven by the diminished catalytic turnover (k_cat_), counterbalanced by an overall conserved binding affinity (as reflected in K_M_) (Figure 4, Table 1). K_aza_ was found to be a kinetically comparable analogue to natural lysine, in line with recent data on KMTs [20]. Despite the introduction of a geometric constraint in lysine side chain, in terms of a forced “zig-zag”-like conformation, the K_E_-containing histone peptide was found to be a slightly worse (3-fold) substrate for KAT8, but still comparatively better than for related KMTs [28]. Given the lack of biostructural information, this finding could be the first indirect molecular clue of lysine’s side chain preferential conformation in the KAT8 active site, although limited structural data showed that other KATs bind lysine in the “non zig-zag” side chain orientation (Figure 1b, Figures S22 and S23). The case was different for βhK, which still displayed a reduced catalytic activity, despite a slightly more favourable K_M_ value, as a possible result of improved backbone interactions with KAT8.

### 2.4. Anaysis of Peptides Inhibitory Profile

Finally, we evaluated the inhibitory potential of the histone peptides that were not observed as KAT8 substrates. The incubation of an equimolar amount of H4K16 and inhibitor peptide H4K*16 (100 µM) with KAT8 revealed that none of the histone peptides exhibited significant inhibition potency (Figure S24). This result is in line with previous findings, most likely dictated by the low binding affinity of histone peptides towards KAT8 [10,29].

## 3. Materials and Methods

### 3.1. Synthesis and Purification of Histone Peptides

All the histone peptides were manually synthesised with standard Fmoc-SPPS chemistry on a 0.05 mmol scale on Wang resin (0.87 g/mmol loading capacity, 100–200 mesh) as previously described [10,11]. Briefly, amino acids (3.0 eq) were coupled with HOBt (3.6 eq) and DIC (3.3 eq) in DMF (final volume 2 mL) for 45 min to 2 h at room temperature. Incorporation of unnatural lysine analogues (1.3 eq) was performed overnight at room temperature, with HOBt (1.56 eq) and DIC (1.43 eq). Incorporation of K_Nme_ and Abg (3.0 eq) was achieved by coupling with HATU (2.9 eq) and DIPEA (5 eq) for 4 h. Fmoc deprotection was achieved with 20% of piperidine in DMF (*v*/*v*) in 20 min. The qualitative Kaiser Test was used to monitor the coupling and deprotection steps. DMF washes (bubbling 3 × 2 min with N_2_) were carried out after both deprotection and coupling steps. Upon the incorporation of the last amino acid, the peptides were Fmoc deprotected, dried over Et_2_O and under vacuum, and subsequently cleaved with a cleavage cocktail (95% TFA, 2.5% TIPS, 2.5% Milli-Q water, 2–3 mL), shaking for 4 h at room temperature. Crude peptides were precipitated on cold Et_2_O (− 20 °C), lyophilised and purified by preparative RP-HPLC. The eluent system constituted of a multistep gradient of Solvent B (0.1% TFA in CH_3_CN) in Solvent A (0.1% TFA in Milli-Q water). Mobile phase gradient: 0–3 min (3%), 3–15 min (3–20%), 15–20 min (20–35%), 20–22 min (35–100%), 22–25 (100%), 25–27 min (100–3%) and 27–30 min (3%) for re-equilibration of the column. Pooled pure fractions were freeze-dried, yielding the target peptides as white solids. The purity of the synthesised peptides was then assessed via MALDI TOF–MS and analytical RP-HPLC (Figures S1–S16).

### 3.2. Expression and Purification of KAT8

His-tagged human KAT8 catalytic domain (residues 125–458) was expressed and purified as previously described [10,11].

### 3.3. MALDI-TOF MS Enzymatic Assays

KAT8 enzymatic activity towards H4K*16 histone peptides was measured at different time points under standard conditions (2 µM KAT8, 100 µM peptide, 300 µM AcCoA) in reaction buffer (50 mM HEPES, 0.1 mM EDTA, 1 mM DTT, pH = 8.0) as previously described [10,11] The reactions were performed in a final volume of 50 µL and shaken at 37 °C, using a Thermomixer C (750 rpm). KAT8 activity was stopped at a given time point by the addition of TFA 10% in Milli-Q water, and reactions were analysed by MALDI-TOF MS employing α-Cyano-4-hydroxycinnamic acid (CHCA) matrix.

### 3.4. MALDI-TOF MS Kinetics Assays

Histone peptides kinetics evaluation was carried out with a MALDI-TOF MS assay under steady-state conditions as previously described [10,11]. Briefly, dilution series of H4K*16 peptides (1050 or 700–0 µM) were incubated with AcCoA (100 µM; > 5 x K_M_ value of AcCoA) and the reactions were started by the addition of KAT8 (570 nM) in kinetic buffer (50 mM HEPES, 0.1 mM EDTA, 0.01% TRITON-X, pH = 7.4). AcCoA stock solutions in Milli-Q water were calibrated using a NanoDrop 2000 spectrophotometer (Thermo Scientific, Waltham, MA, USA), with molar extinction coefficient ε_260nm_ = 16,400 M^−1^ cm^−1^. Reactions were incubated for 20 min at 37 °C, shaken at 750 rpm and quenched with TFA 10% in Milli-Q water at different time points within the linear production of acetylated peptides. Quantification of the acetylated peptide produced was extrapolated, at any concentration point, with FlexAnalysis software, considering all the ionic species. Kinetic parameters were obtained by fitting V_0_ values and histone peptide concentrations to the Michaelis–Menten equation using the GraphPad Prism 5 software. Kinetic experiments were carried out in replicates (n = 2 or 3) and final values are reported as the mean value ± SD.

### 3.5. Inhibition Assays

Equimolar amounts of the H4K16 histone peptide and H4K*16 histone peptide inhibitor (1:1; 100 µM) were incubated in the presence of AcCoA (300 µM) in the reaction buffer (50 mM HEPES, 0.1 mM EDTA, 1 mM DTT, pH = 8.0). The reactions were started by adding KAT8 (200 nM) to the mixture, and were carried out at 37 °C for 30 min, after which they were quenched by the addition of 10% TFA in Milli-Q water and analysed by MALDI-TOF MS. Every experiment was carried out in replicates (n = 2) and normalised to the positive control reaction. Data are shown as the mean value ± SD.

## 4. Conclusions 

Due to the lack of accurate crystallographic information, the usage of unnatural amino acids has been established to be one of the most powerful experimental tools to obtain essential biostructural information regarding novel enzyme catalysis and active site dynamics. The analysis of the chemical basis that regulates the activity of important epigenetic enzymes has recently emerged and consolidated an uprising field in the area of chemical biology, with remarkable efforts especially invested in the study of KMTs [20,21,24,27,28]. Despite the great biomedical relevance, the biocatalytic potential of KATs is yet to be unravelled. Our enzymatic studies on a chemically and structurally diverse panel of lysine mimics demonstrate that KAT8 has a broader substrate scope beyond lysine. The identification of novel KAT8 substrates, bearing lysine analogues with modified nucleophilic properties (K_aza_ and K_N_), geometric constrains (K_E_), and altered main chain (βhK), shed light on the molecular prerequisites underlying histone acetylation transfer reaction. Overall, our results contribute to a better understanding of biomedically important KAT enzymes, as well as lay the foundation for the rational development of new therapeutic agents for KAT8.

## Figures and Tables

**Table 1:** Kinetics parameters for the KAT8-catalysed acetylation of H4K*16 histone peptides.

H4 Peptide	k_cat_^app^ (min^−1^)	K_M_^app^ (μM)	k_cat_^app^/K_M_^app^ (mM^−1^ min^−1^)
H4K16	15 ± 0.8	437 ± 107	34
H4K_aza_16	12.4 ± 0.5	405 ± 66	31
H4K_E_16	5.3 ± 0.4	486 ± 124	11
H4βhK16	2.2 ± 0.1	283 ± 67	8

## Data Availability

The data presented in this study are available in Supplementary Materials of this article.

## Supplementary Materials

The following are available online at https://www.mdpi.com/1422-0067/22/2/846/s1.
