# Supplementary material for: Substrate Scope for Human Histone Lysine Acetyltransferase KAT8
Source: Int J Mol Sci. 2021 Jan 15;22(2):846. doi: 10.3390/ijms22020846 (PMC7830570; doi:10.3390/ijms22020846)

Supplementary Information

Substrate Scope for Human Histone Acetyltransferase KAT8

Giordano Proietti[1,2], Yali Wang,[2,3] Chiara Punzo[1] and Jasmin Mecinović[1,2]*

[1] Department of Physics, Chemistry and Pharmacy, University of Southern Denmark, Campusvej 55, 5230 Odense, Denmark.

[2] Institute for Molecules and Materials, Radboud University, Heyendaalseweg 135, 6525 AJ Nijmegen, The Netherlands.

[3] Department of Blood Transfusion, China-Japan Union Hospital, Jilin University, 126 Xiantai Street, Changchun, 130033, P. R. China.

*e-mail: mecinovic@sdu.dk

Table of Contents

1	General experimental	3
2	Synthetic procedures	4
3	List of histone peptides	5
4	Characterization of histone peptides	7
5	Time course plot of KAT8-catalysed acetylation	23
6	MALDI and crystallographic supporting figures	24
7	Inhibition plot	29
8	NMR spectra	30

1. General Experimental

All commercially available reagents were purchased and used without further purification. Unless otherwise noted all the reagents were purchased from Sigma-Aldrich. Unnatural lysine analogs were purchased from either Sigma-Aldrich, Novabiochem, ChemImpex or Iris Biotech GmbH. Reactions were magnetically stirred, and monitoring by thin layer chromatography (TLC) was performed on glass backed silica sheets (Merck Silica Gel 60 F254) and the plates were visualized by UV fluorescence (254 nm) and/or spraying with potassium permanganate (KMnO4) or ninhydrin. ^{1}H NMR and ^{13}C NMR spectra were obtained using a Bruker Avance III 400 MHz. ^{1}H NMR chemical shift values are reported as δ in units of parts per million (ppm) relative to the internal standard tetramethylsilane (TMS, δ = 0 ppm). ^{13}C NMR shifts are reported as δ in units of parts per million (ppm) and the spectra were internally referenced to the residual solvent signal (CHCl3 δ = 77.0 ppm). Coupling constant are reported as *J* values in Hertz (HZ). The following abbreviations were used to explain multiplicities: s = singlet, d = doublet, t = triplet, q = quartet, m = multiplet, br = broad. Histone peptides were purified on a preparative HPLC employing a Phenomenex Gemini-NX 3u C18 110A reversed-phase column (150 × 21.2 mm) with a flow rate of 10 mL/min at 30 °C. Analytical traces were monitored at 215 nm on a Phenomenex Gemini 5 µm C18 110 Å LC column at a flow rate of 1 mL/min. MALDI-TOF MS enzymatic analysis was performed by mixing aliquotes of the reaction mixture with the α-Cyano-4-hydroxycinnamic acid (CHCCA) matrix in 1:1 MQ and ACN (0.1% TFA) and MALDI-TOF MS spectra were recorded by a UltrafleXtreme-II tandem mass spectrometer (Bruker, Billerica, MA, USA) employing a MTP 384 polished steel target.

2. Synthetic procedures

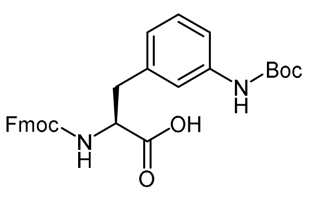

(S)-2-((((9H-fluoren-9-yl)methoxy)carbonyl)amino)-3-(3-((tert-butoxycarbonyl)amino)phenyl)propanoic acid (1). An oven-dried flask equipped with magnetic stirrer was loaded with Pd/C (31 mg, 10 wt. %) under Argon atmosphere. A solution of Fmoc-3-nitro-L-phenylalanine (300 mg, 0.7 mmol) in 5 mL of EtOAc was added to the flask, and the volume was brought to 80 mL. The Argon stream was replaced by H_2 gas (1 atm), the flask back-purged 3 times with a syringe and the reaction mixture was left stirring and monitored through TLC, the reduction product being displayed with ninhydrin spray (10% MeOH in DCM; Rf = 0.38). After 5 days, the off-white precipitate which appeared was dissolved by the addition of MeOH (30 mL) and the mixture was filtered through celite and concentrated in vacuo. The resulting white-off solid was taken up in a 1:1 solution of water/dioxane (35 mL) and $NaHCO_3$ (123 mg, 1.47 mmol, 2.1 eq) was added. The reaction mixture was then cooled down to 0 °C and followed by the addition of Di-tert-butyl dicarbonate (229 mg, 1 mmol, 1.5 eq.) over a period of 10 minutes. After 48 hours additional Di-tert-butyl dicarbonate (229 mg, 1 mmol, 1.5 eq.) was added. After additional 48 hours at room temperature, the solvents were removed in vacuo, and the resulting white solid was taken up in EtOAc (15 mL) acidified with diluted 5% HCl (1 ml). and extracted with water (3 x 20 mL). The organic phase was collected and dried over $MgSO_4$, filtered, and concentrated under reduced pressure. The mixture was purified by silica gel column chromatography (5-10% MeOH in DCM) to yield compound **2** as a white solid (225 mg, 64 % over two steps). R*f*: 0.62 (10% MeOH in DCM).^{1}H NMR (400 MHz, DMSO-d_6) δ 9.23 (s, 1H), 7.86 (d, J = 7.5 Hz, 2H), 7.61 (t, J = 6.4 Hz, 2H), 7.43 – 7.35 (m, 3H), 7.31 – 7.22 (m, 4H), 7.13 – 7.07 (m, 2H), 6.86 (d, J = 7.5 Hz, 1H), 4.23 (dd, J = 9.6, 6.9 Hz, 1H, α-CH), 4.16 (t, J = 6.8 Hz, 2H, Fmoc-CH), 4.13 – 4.02 (m, 2H, Fmoc-CH_2), 3.09 (dd, J = 13.9, 4.3 Hz, 1H, β-CH), 2.83 (dd, J = 13.6, 9.1 Hz, 1H, β-CH), 1.43 (s, 9H, Boc-CH_3). ^{13}C NMR (101 MHz, DMSO) δ 174.58, 155.48, 152.68, 140.56, 139.15, 127.92, 127.44, 126.95, 125.08, 123.09, 119.92, 119.06, 116.02, 78.66, 65.42, 56.46, 46.60, 37.45, 28.05. ESI-MS $[M+Na]^+$: 525.2 m/z.

3. List of histone peptides

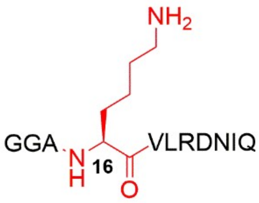

H4K16

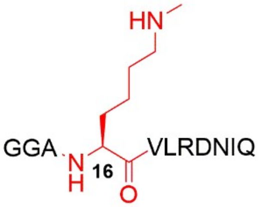

H4Kme16

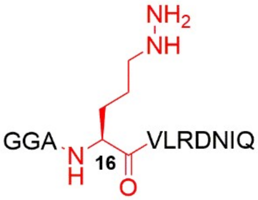

H4K$_{aza}$16

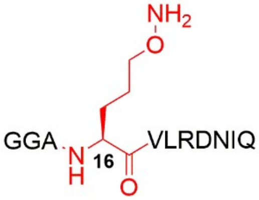

H4K$_{oxy}$16

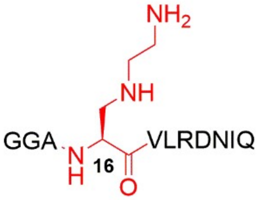

H4K$_{N}$16

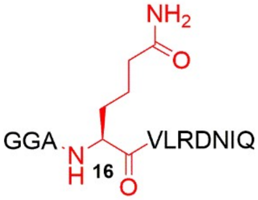

H4hGln16

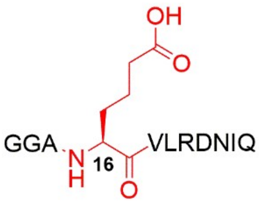

H4K$_{COOH}$16

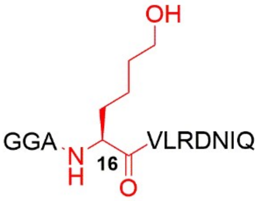

H4K$_{OH}$16

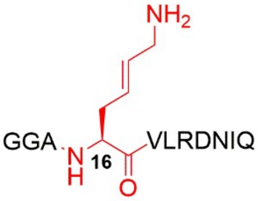

H4K$_{E}$16

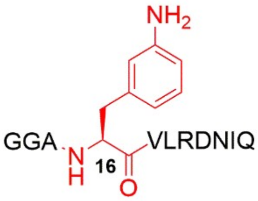

H4F$_{3a}$16

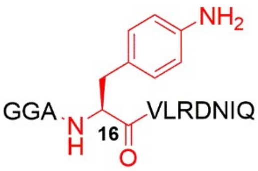

H4F$_{4a}$16

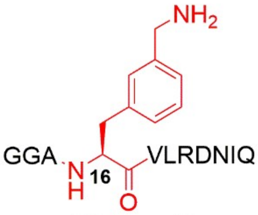

H3F$_{3me\text{-}a}$16

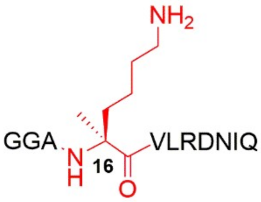

H4K$_{CMe}$16

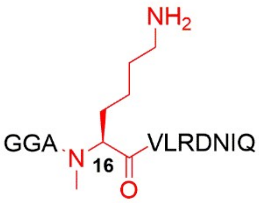

H4K$_{NMe}$16

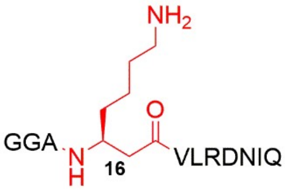

H4β-hK16

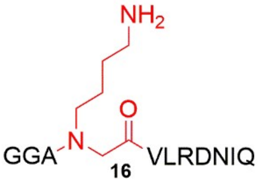

H4Abg16

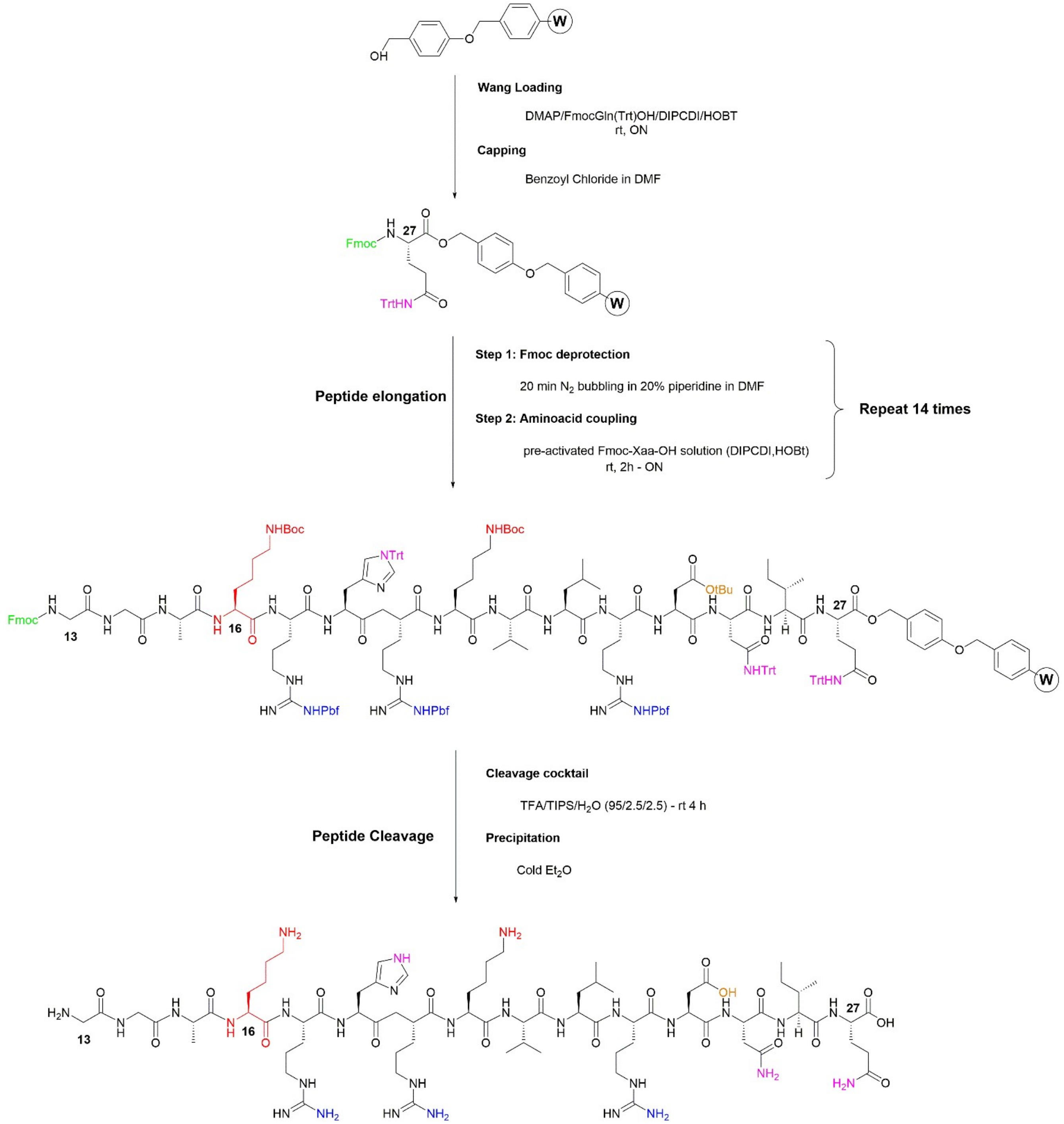

Scheme S1. Solid-phase synthesis of the histone peptide H4K16 (residues 13-27). The same synthetic route was used for synthesis of histone peptides that possess lysine analogues at position 16 of histone 4.

4. Characterization of histone peptides

A)

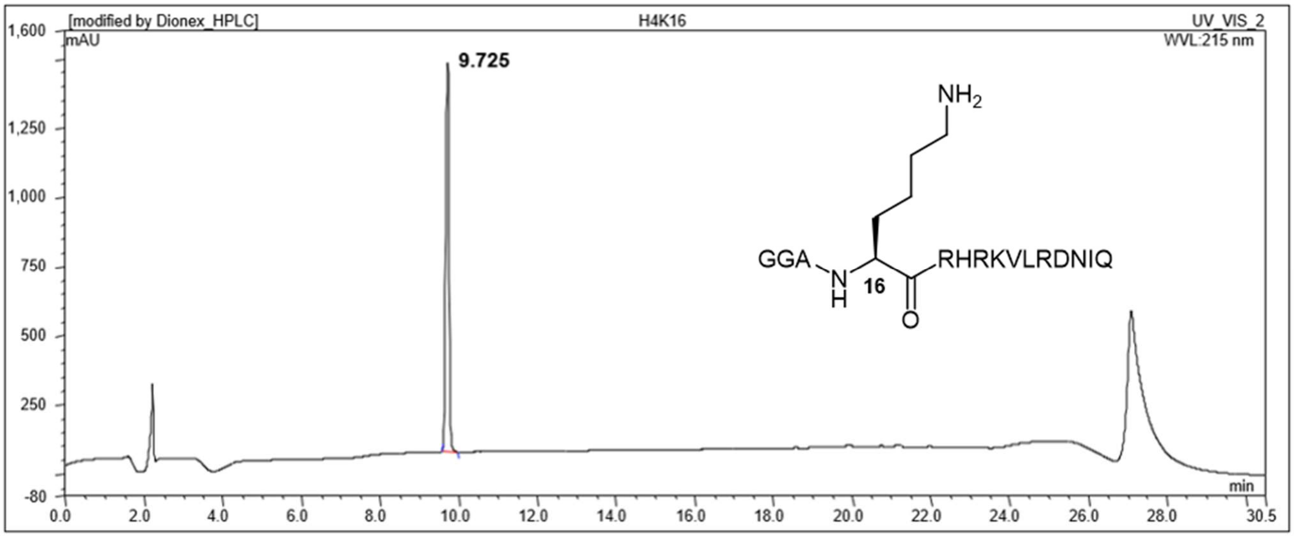

B)

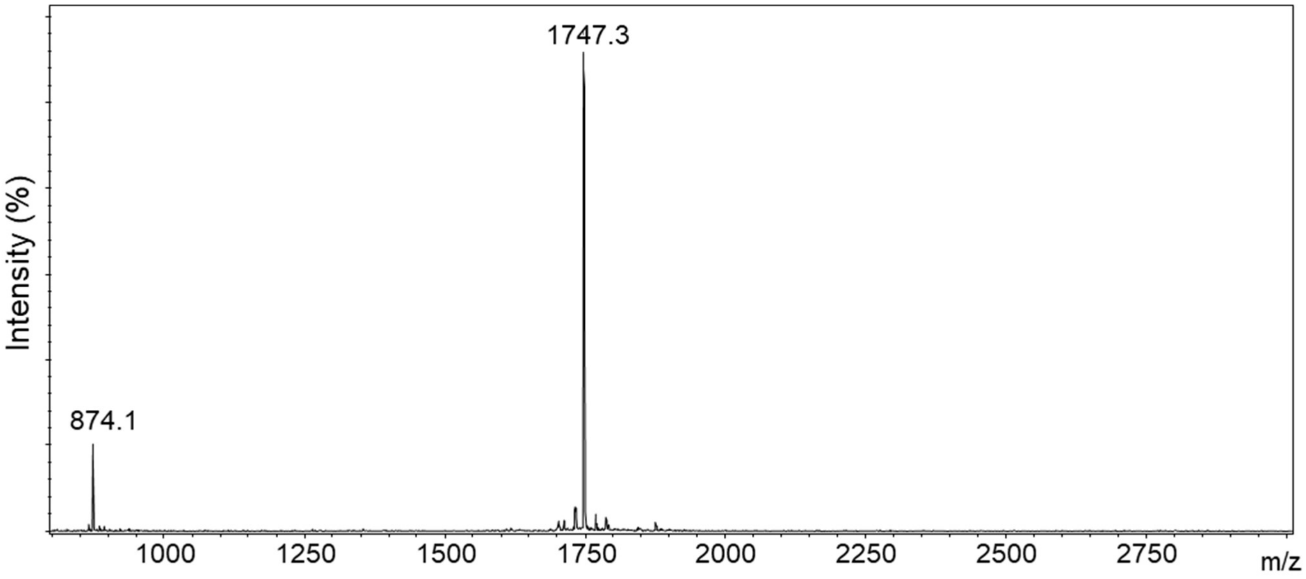

Figure S1. A) Analytical HPLC of the H4K16 peptide after RP-HPLC purification. **B)** MALDI-TOF MS spectra of the purified H4K16 peptide.

A)

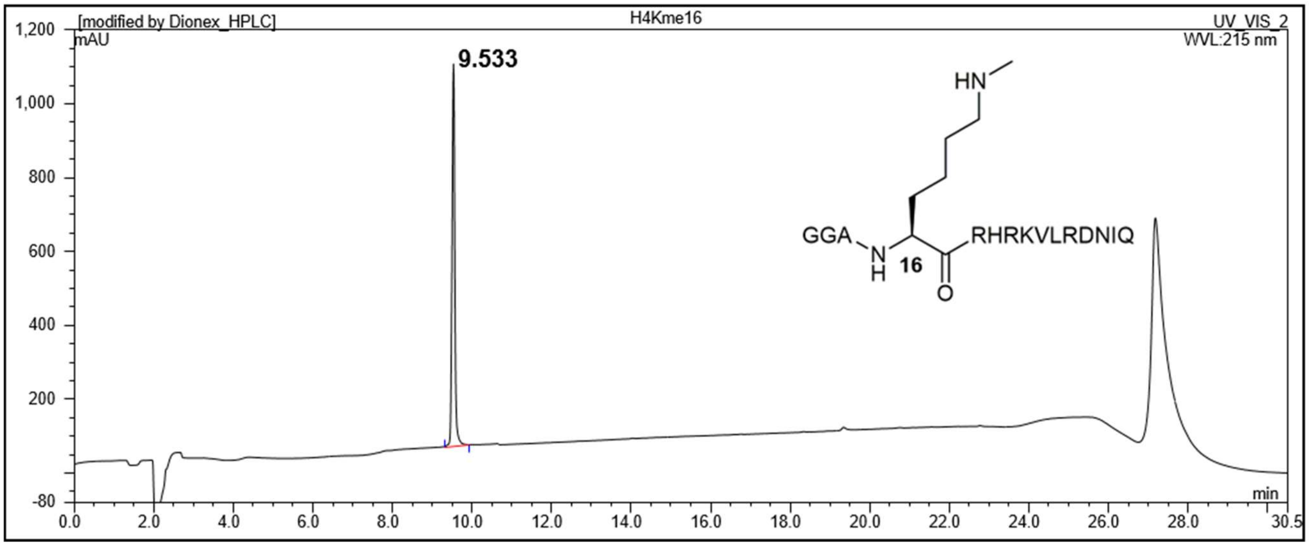

B)

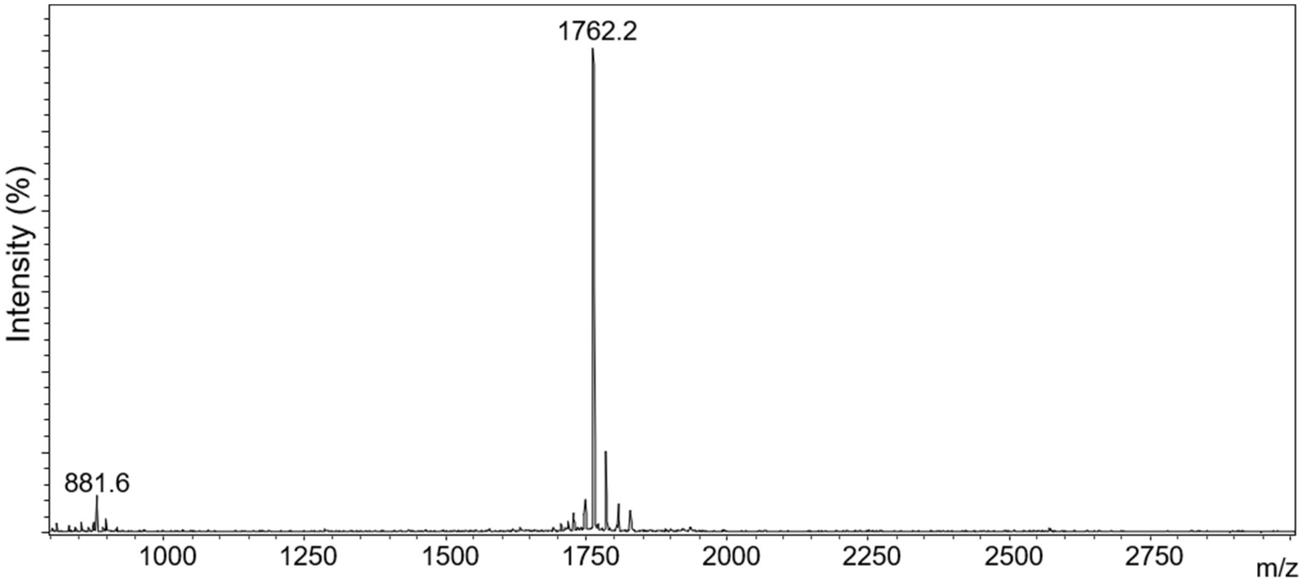

Figure S2. A) Analytical HPLC of the H4Kme16 peptide after RP-HPLC purification. **B)** MALDI-TOF MS spectra of the purified H4Kme16 peptide.

A)

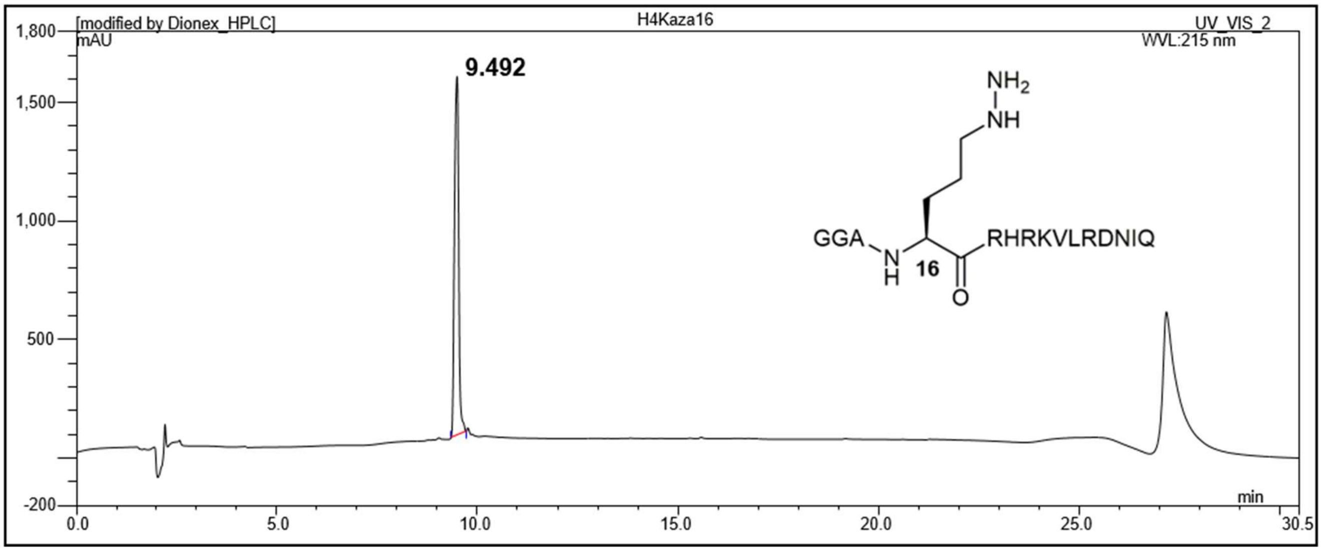

B)

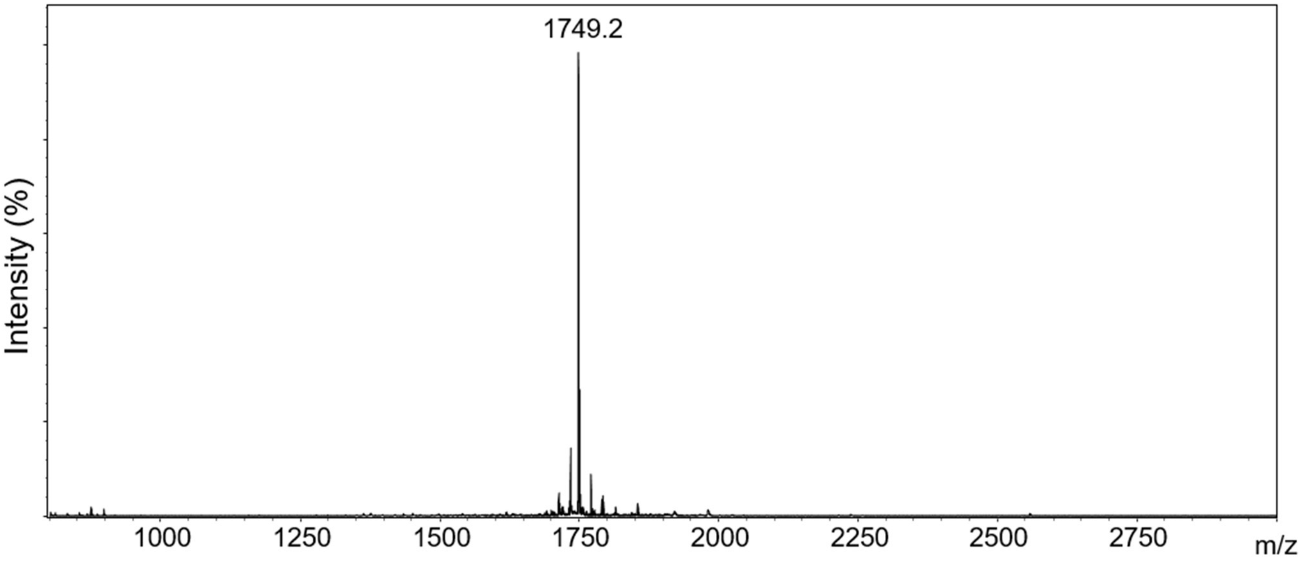

Figure S3. A) Analytical HPLC of the H4K$_{aza}$16 peptide after RP-HPLC purification. **B)** MALDI-TOF MS spectra of the purified H4K$_{aza}$16 peptide.

A)

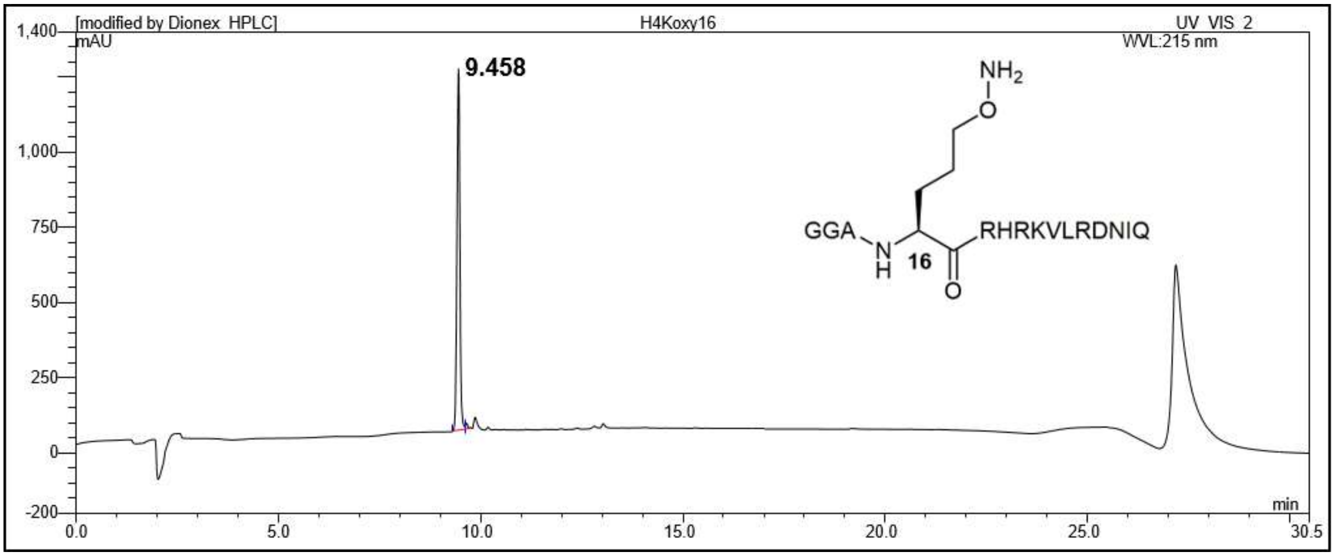

B)

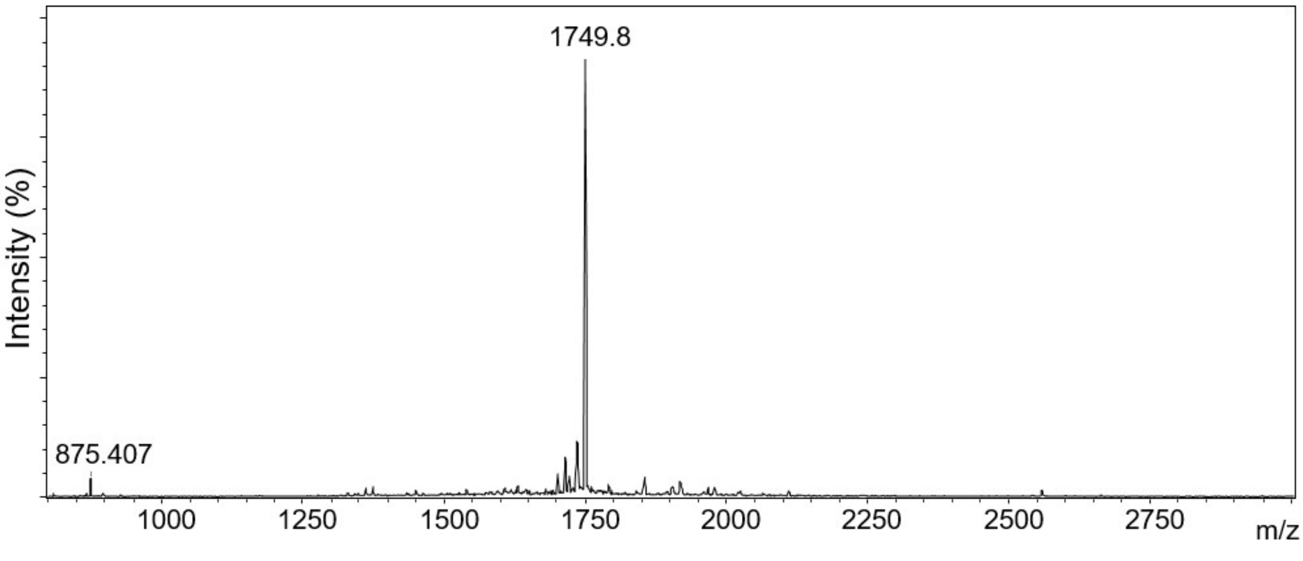

Figure S4. A) Analytical HPLC of the $H4K_{oxy}16$ peptide after RP-HPLC purification. **B)** MALDI-TOF MS spectra of the purified $H4K_{oxy}16$ peptide.

A)

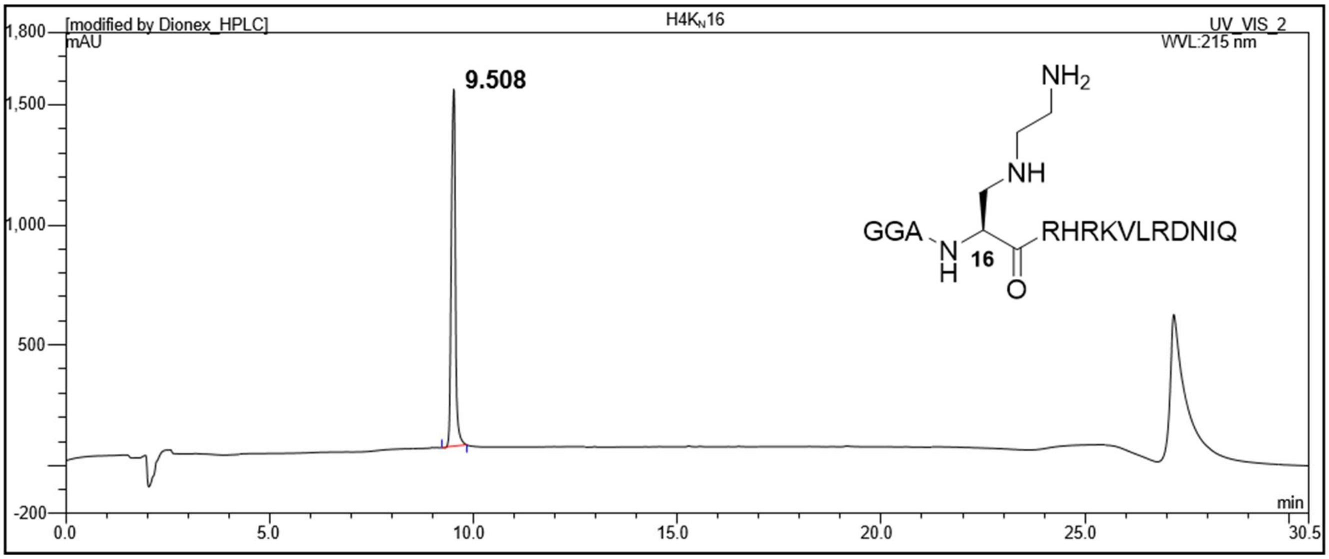

B)

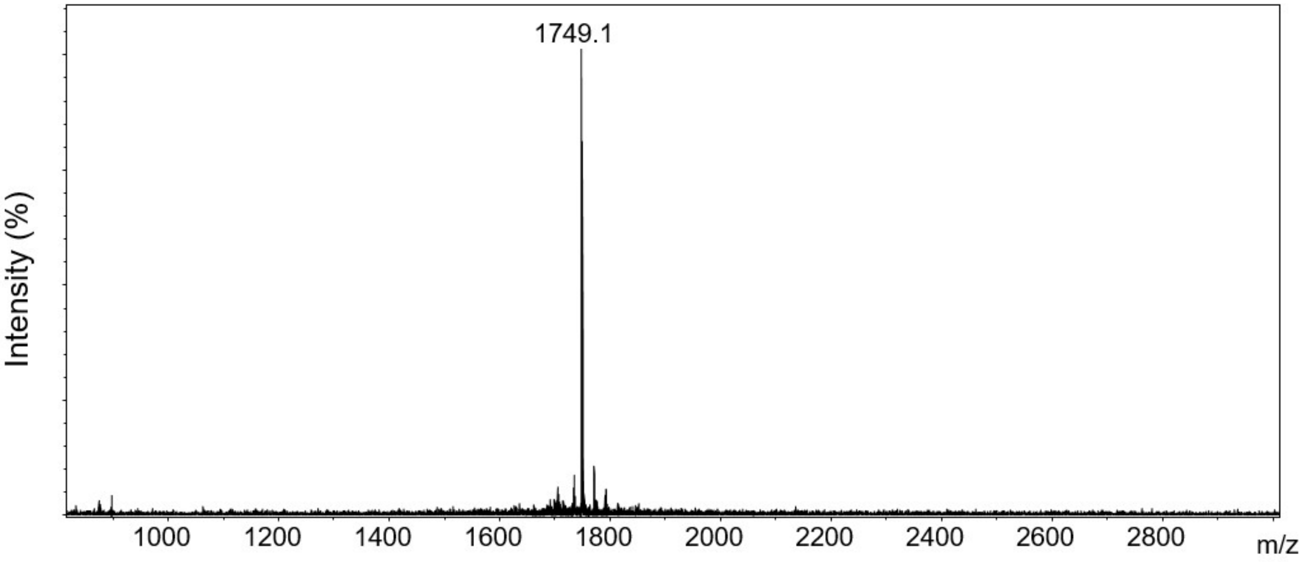

Figure S5. A) Analytical HPLC of the H4K$_N$16 peptide after RP-HPLC purification. **B)** MALDI-TOF MS spectra of the purified H4K$_N$16 peptide.

A)

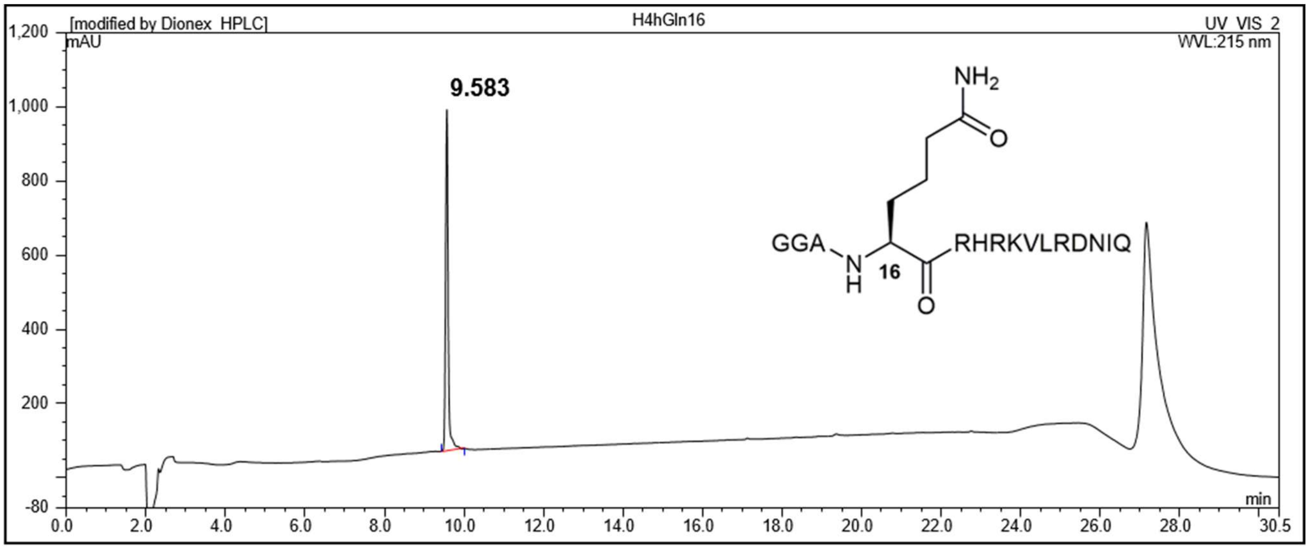

B)

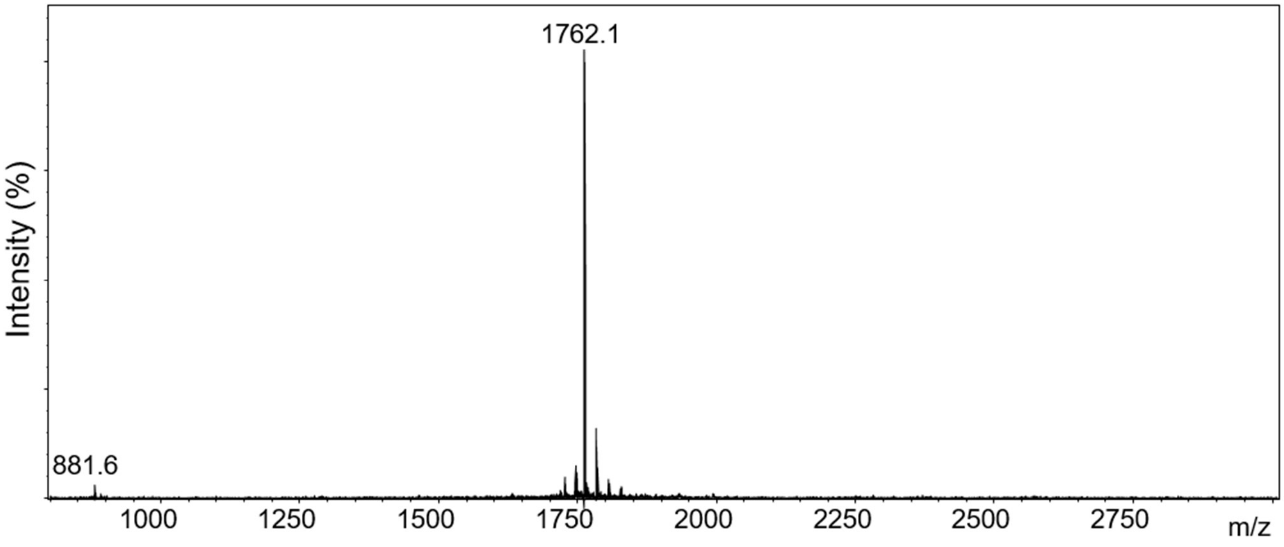

Figure S6. A) Analytical HPLC of the H4hGln16 peptide after RP-HPLC purification. **B)** MALDI-TOF MS spectra of the purified H4hGln16 peptide.

A)

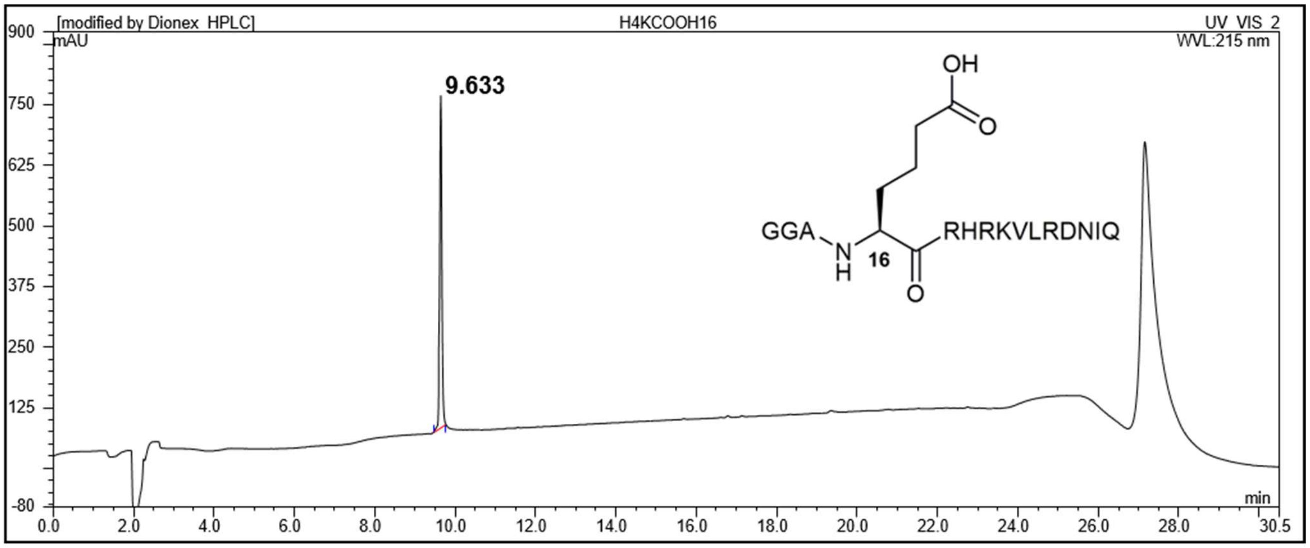

B)

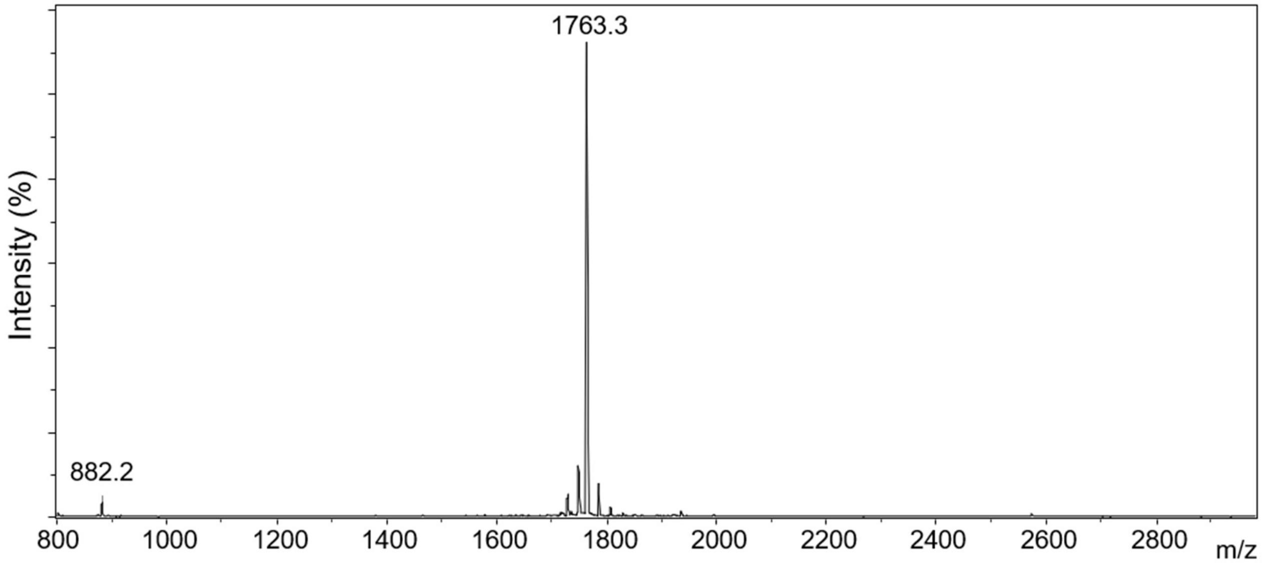

Figure S7. A) Analytical HPLC of the H4K$_{COOH}$16 peptide after RP-HPLC purification. **B)** MALDI-TOF MS spectra of the purified H4K$_{COOH}$16 peptide.

A)

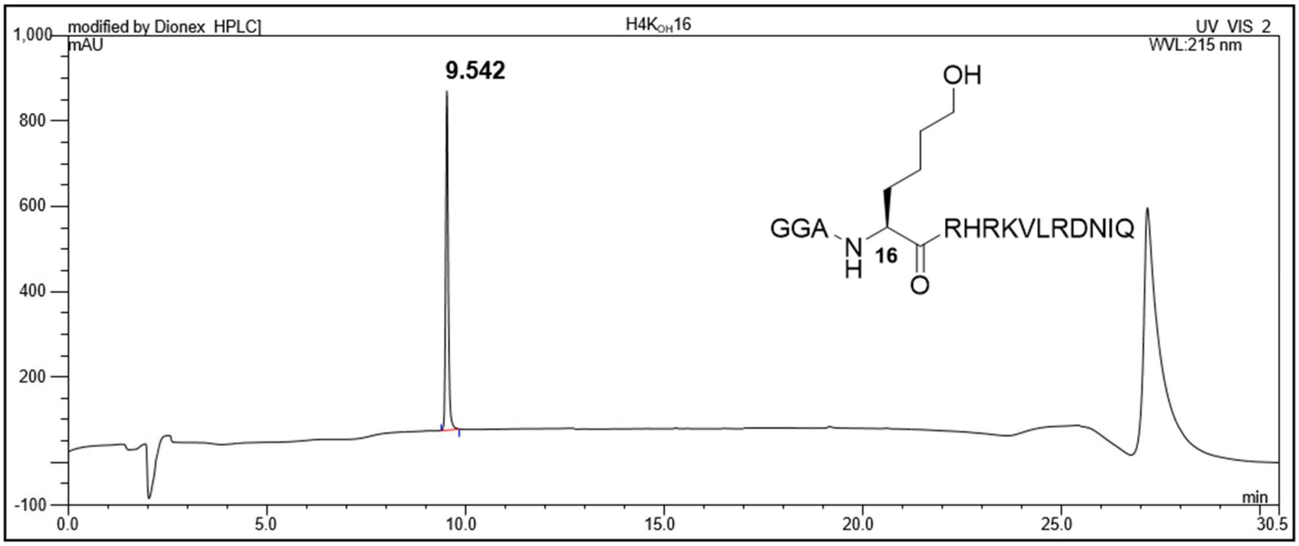

B)

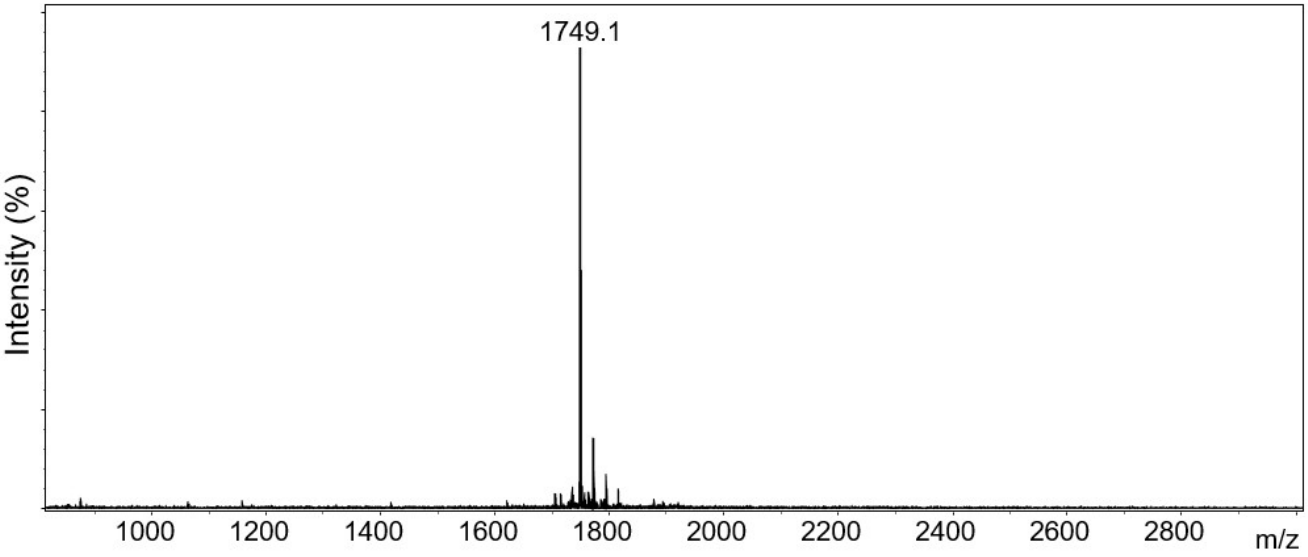

Figure S8. A) Analytical HPLC of the H4K$_{OH}$16 peptide after RP-HPLC purification. **B)** MALDI-TOF MS spectra of the purified H4K$_{OH}$16 peptide.

A)

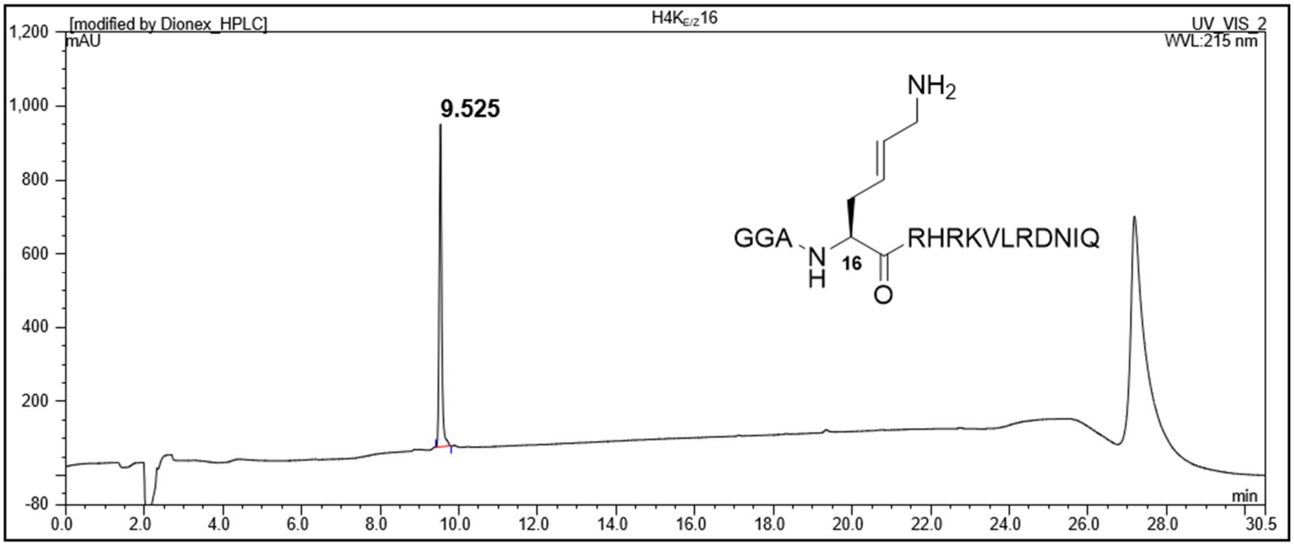

B)

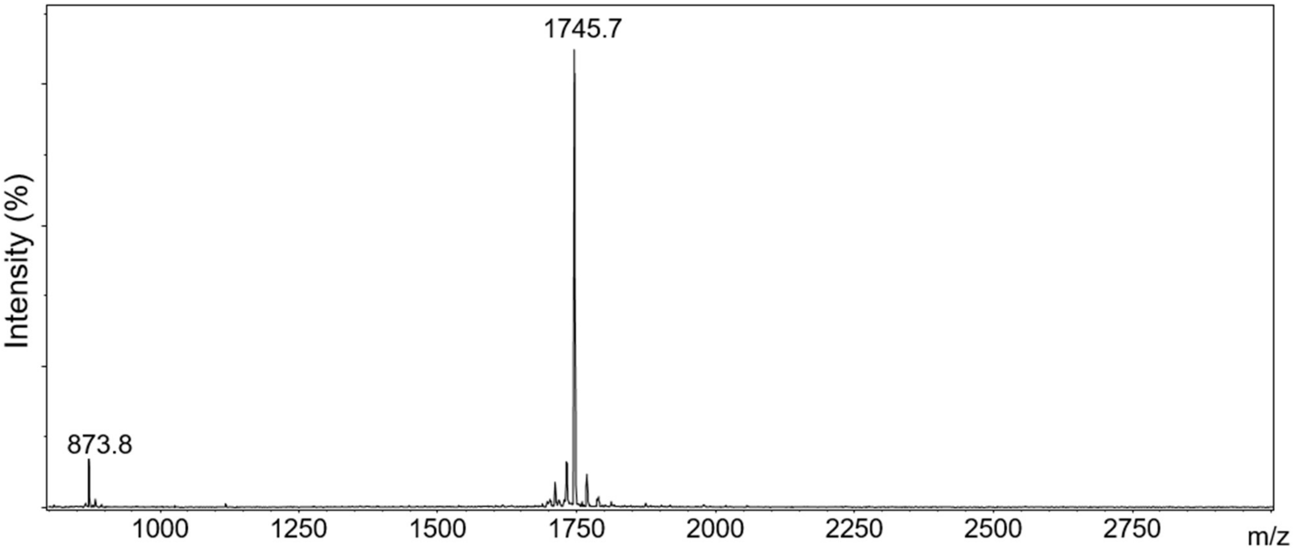

Figure S9. A) Analytical HPLC of the H4K_E16 peptide after RP-HPLC purification. **B)** MALDI-TOF MS spectra of the purified H4K_E16 peptide.

A)

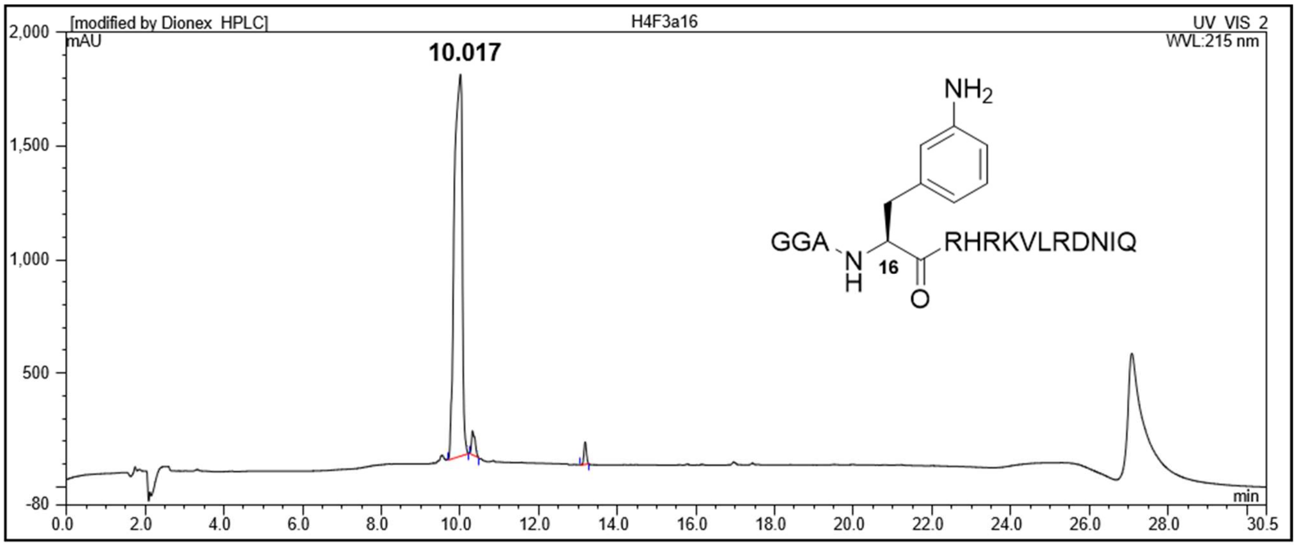

B)

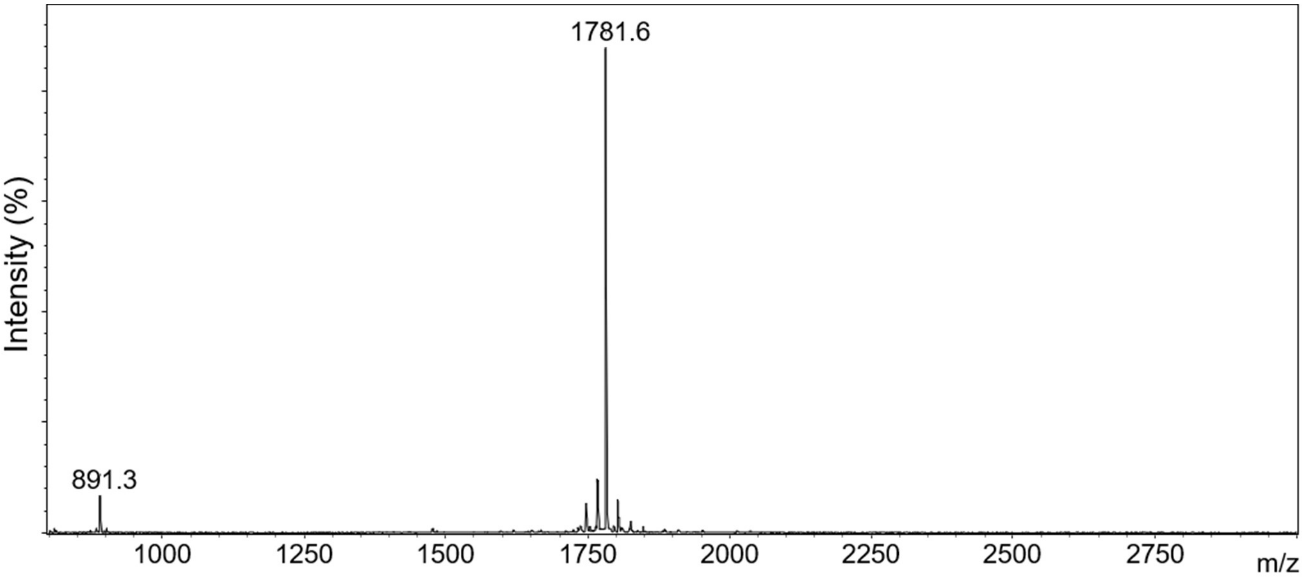

Figure S10. A) Analytical HPLC of the H4K$_{3a}$16 peptide after RP-HPLC purification. **B)** MALDI-TOF MS spectra of the purified H4K$_{3a}$16 peptide.

A)

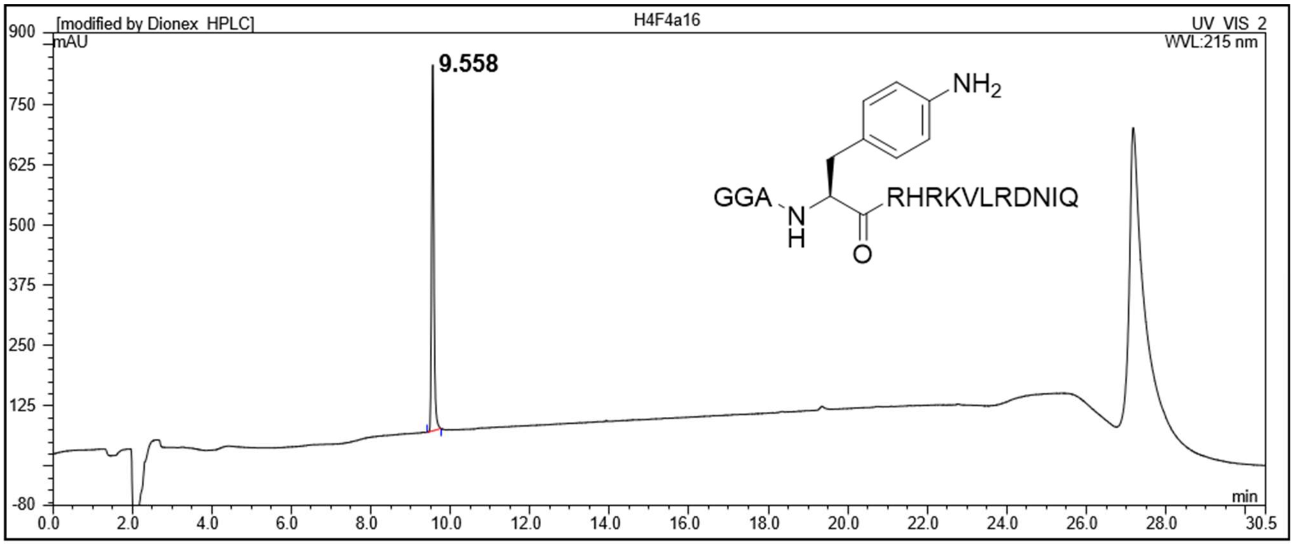

B)

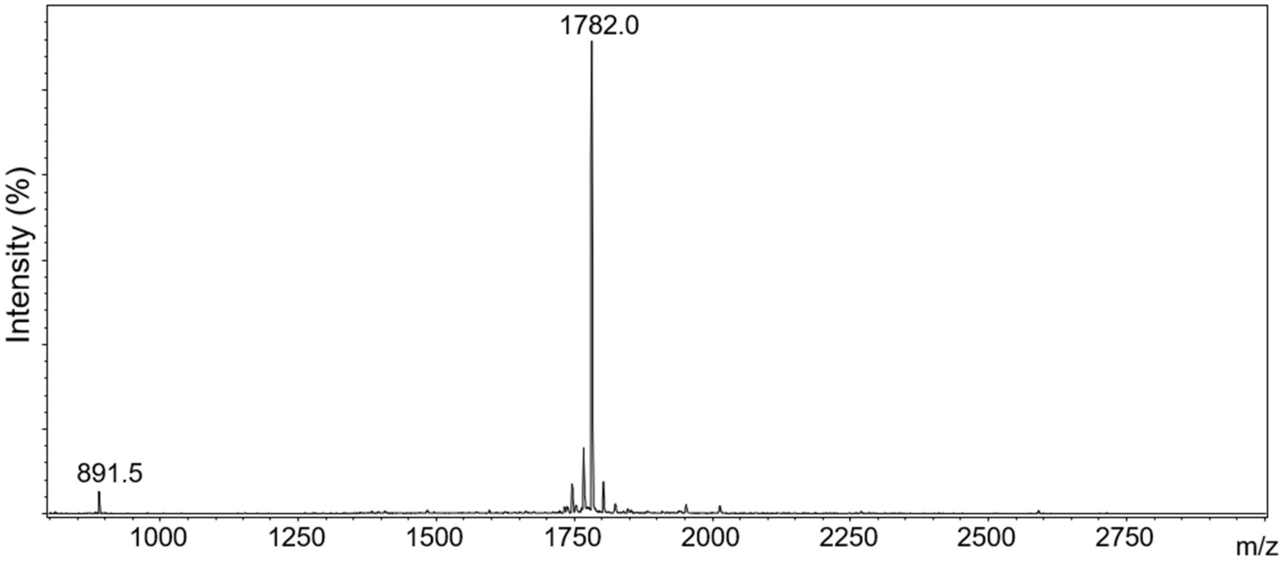

Figure S11. A) Analytical HPLC of the H4F$_{4a}$16 peptide after RP-HPLC purification. **B)** MALDI-TOF MS spectra of the purified H4F$_{4a}$16 peptide.

A)

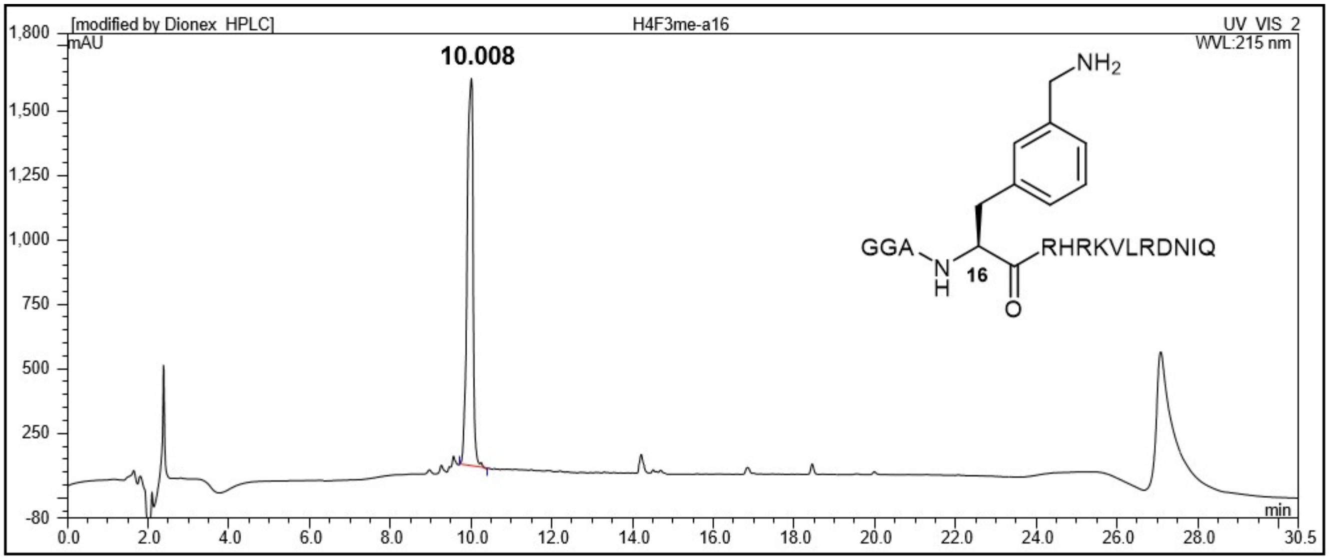

B)

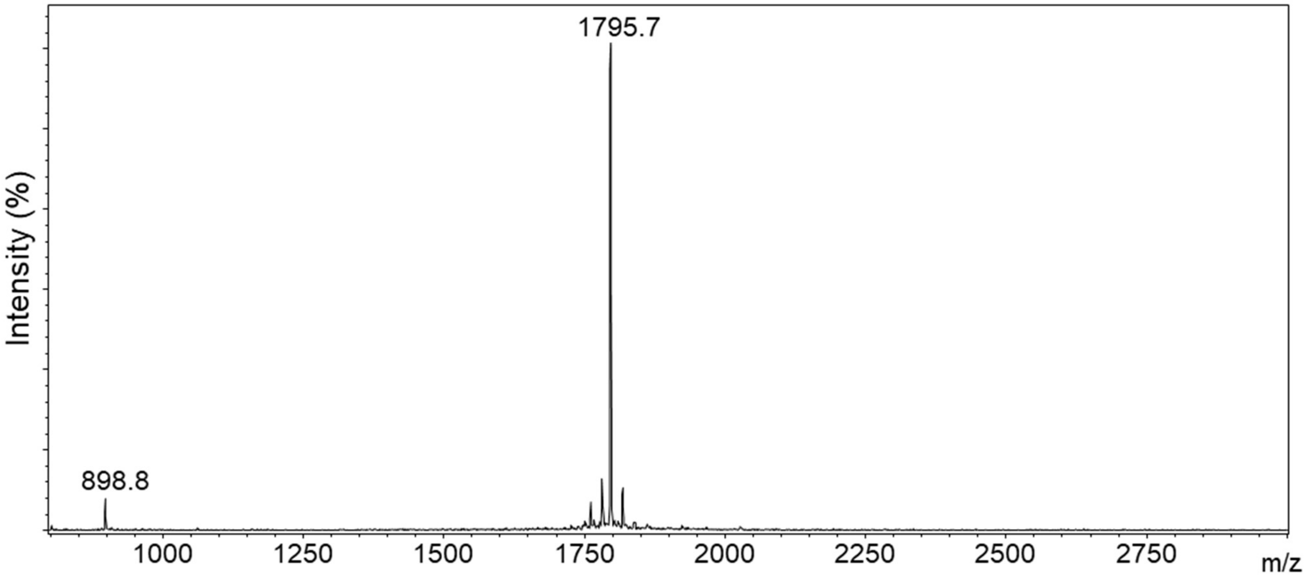

Figure S12. A) Analytical HPLC of the H4$F_{3a\text{-}me}$16 peptide after RP-HPLC purification. **B)** MALDI-TOF MS spectra of the purified H4$F_{3a\text{-}me}$16 peptide.

A)

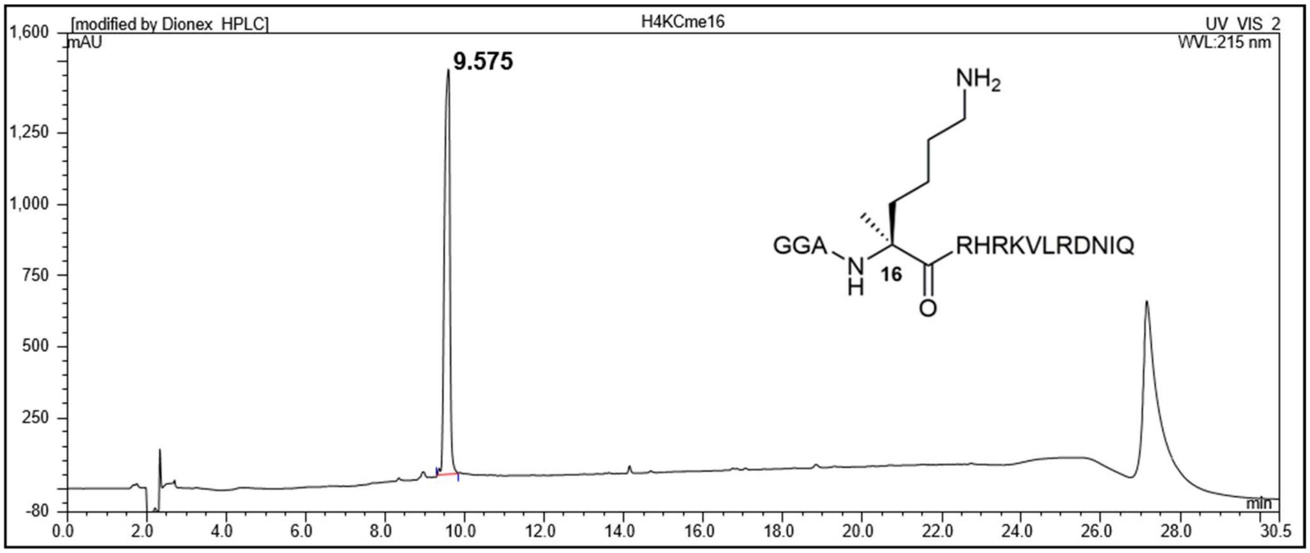

B)

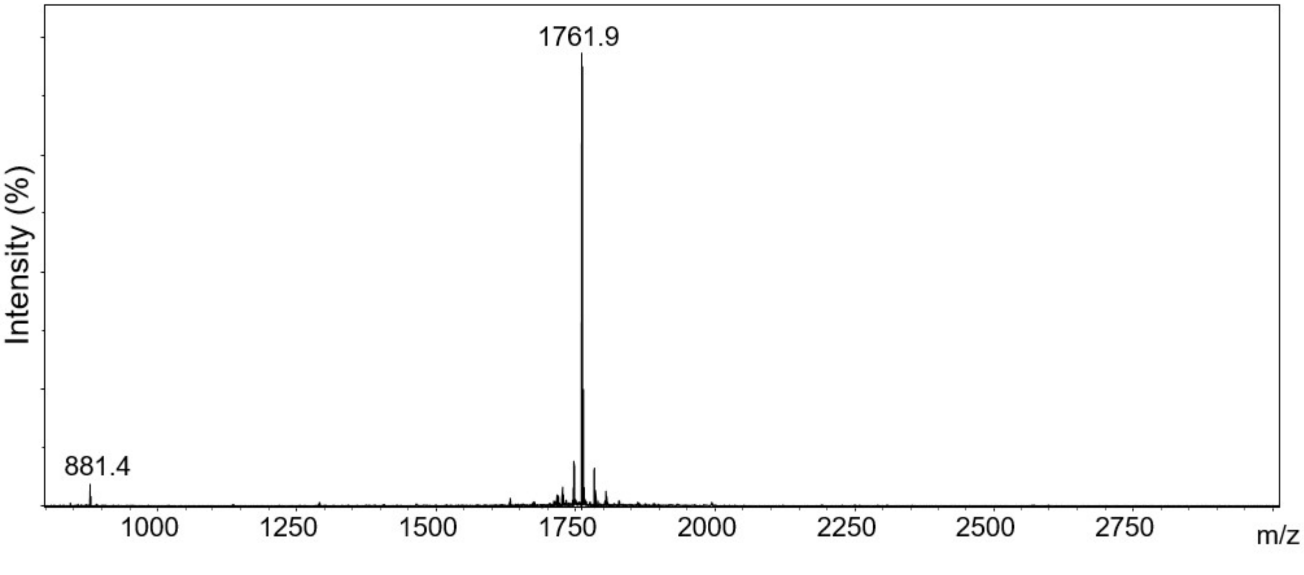

Figure S13. A) Analytical HPLC of the H4K$_{CMe}$16 peptide after RP-HPLC purification. **B)** MALDI-TOF MS spectra of the purified H4K$_{CMe}$16 peptide.

A)

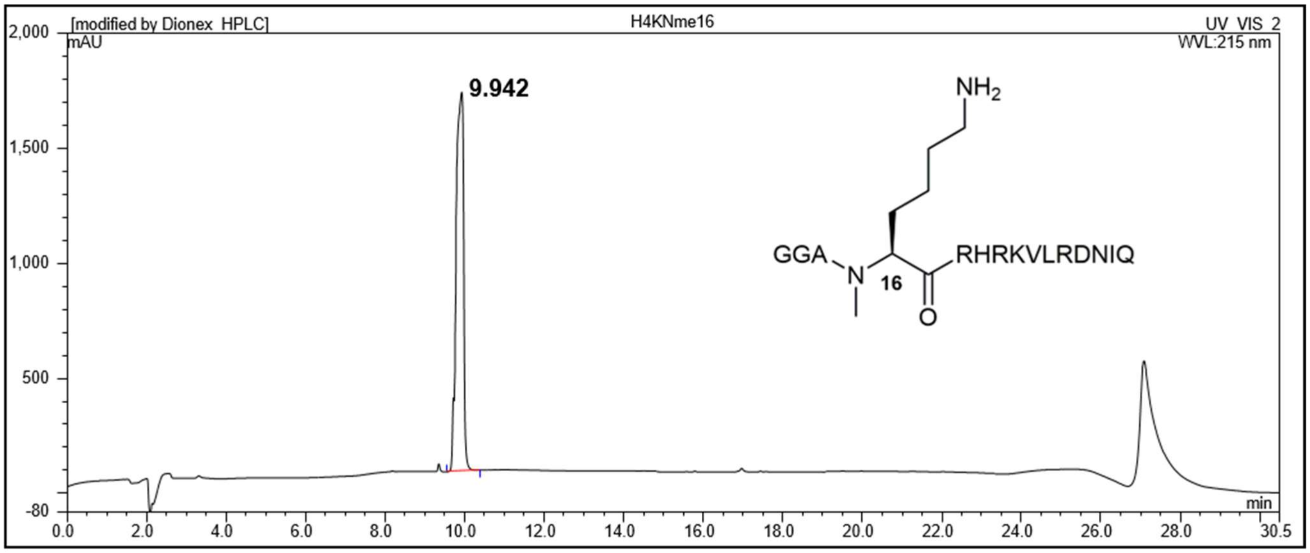

B)

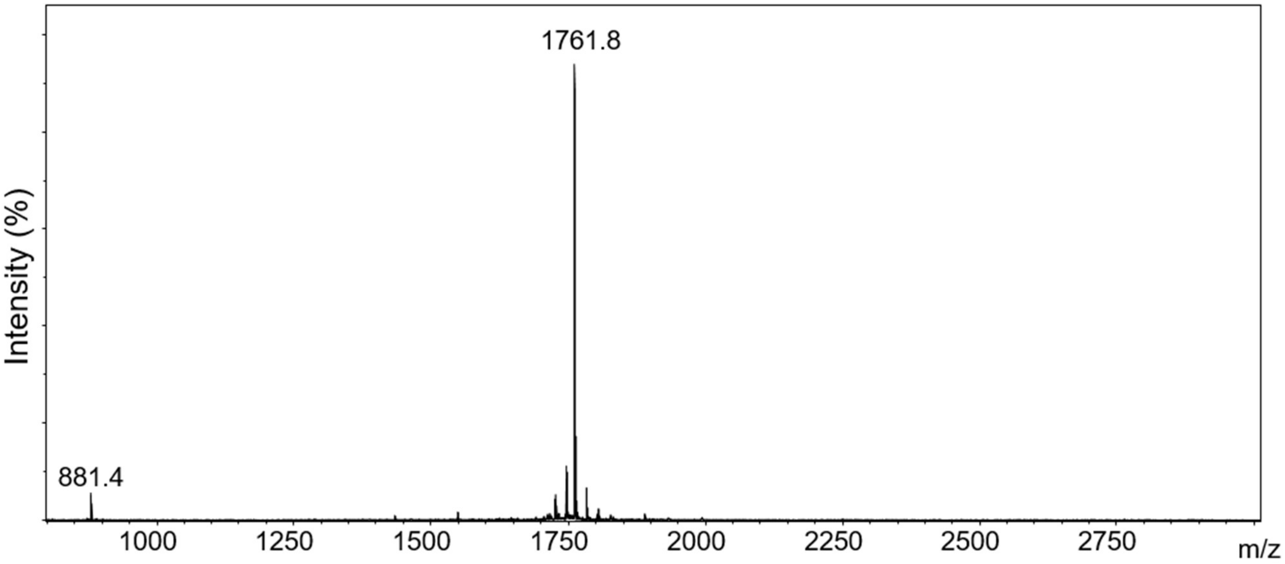

Figure S14. A) Analytical HPLC of the H4K$_{NMe}$16 peptide after RP-HPLC purification. **B)** MALDI-TOF MS spectra of the purified H4K$_{NMe}$16 peptide.

A)

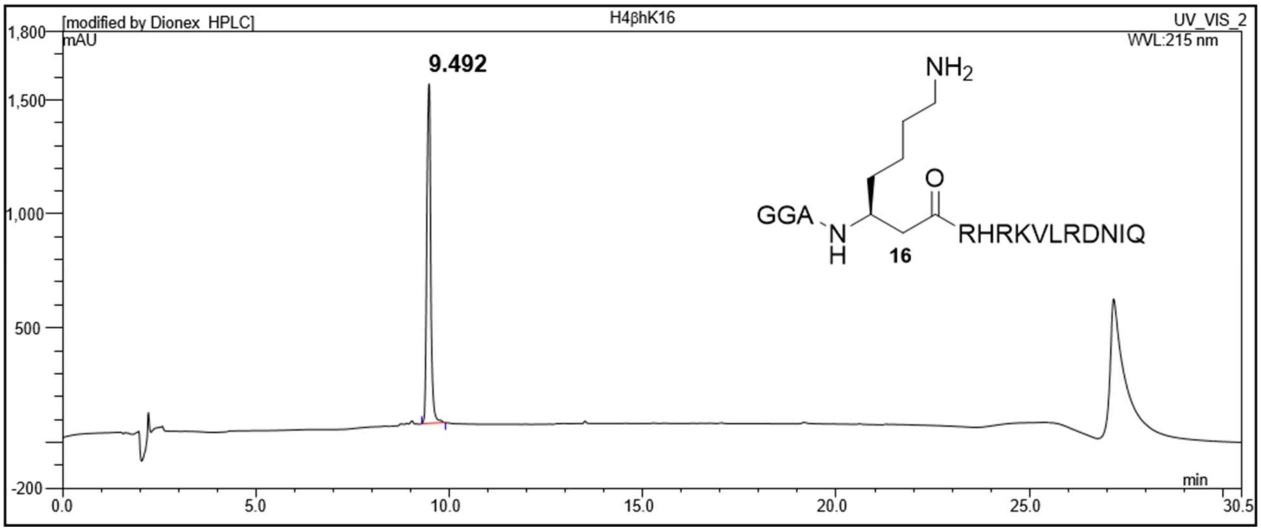

B)

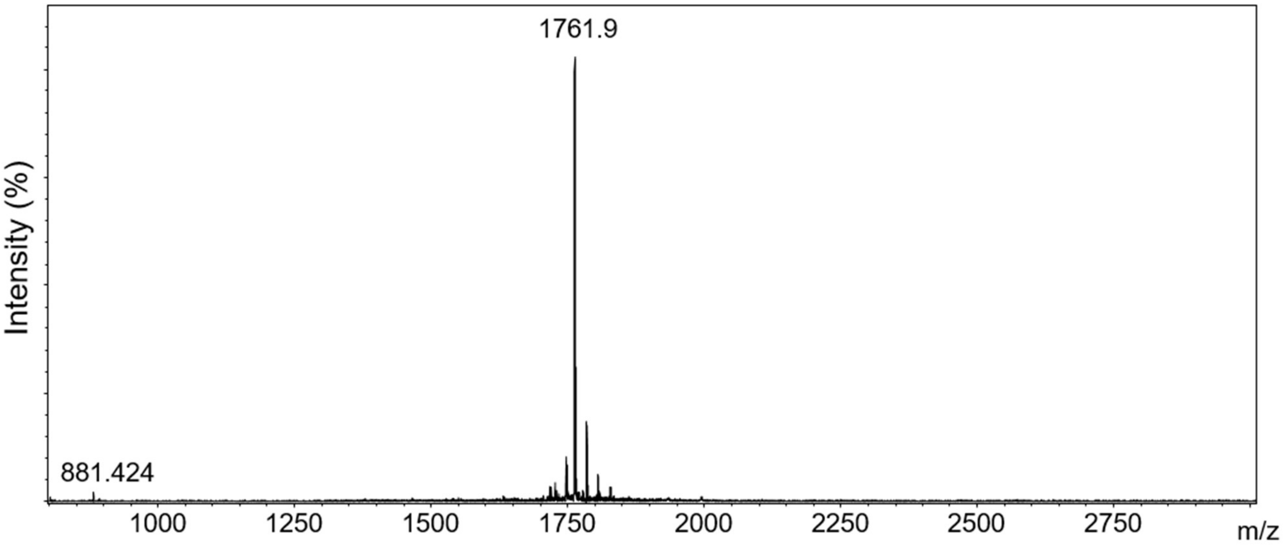

Figure S15. A) Analytical HPLC of the H4βhK16 peptide after RP-HPLC purification. **B)** MALDI-TOF MS spectra of the purified HβhK16 peptide.

A)

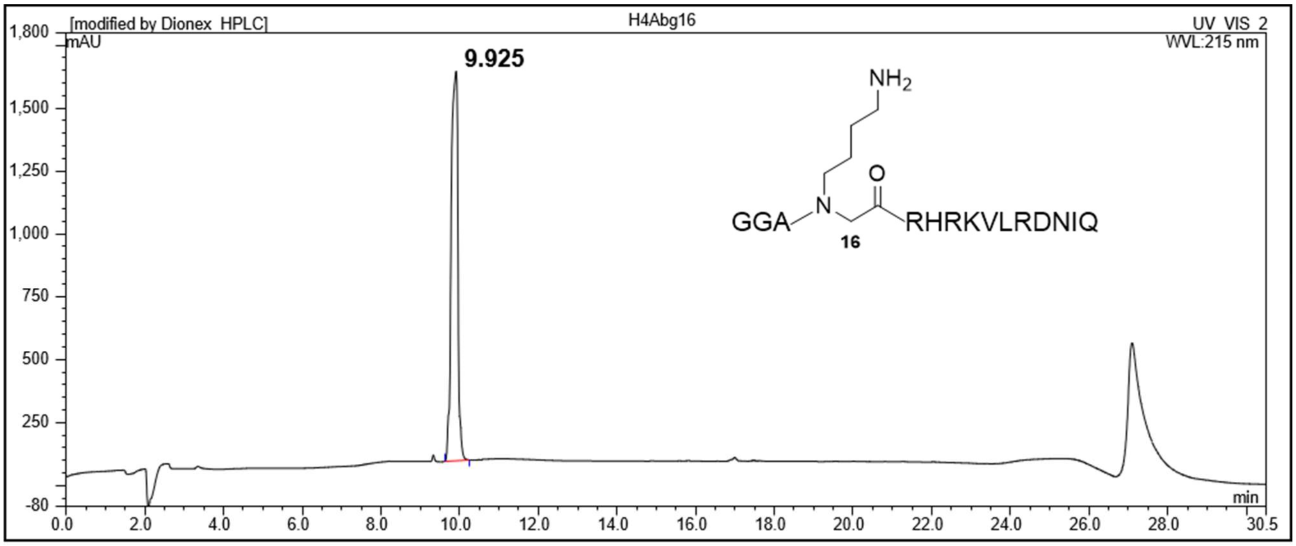

B)

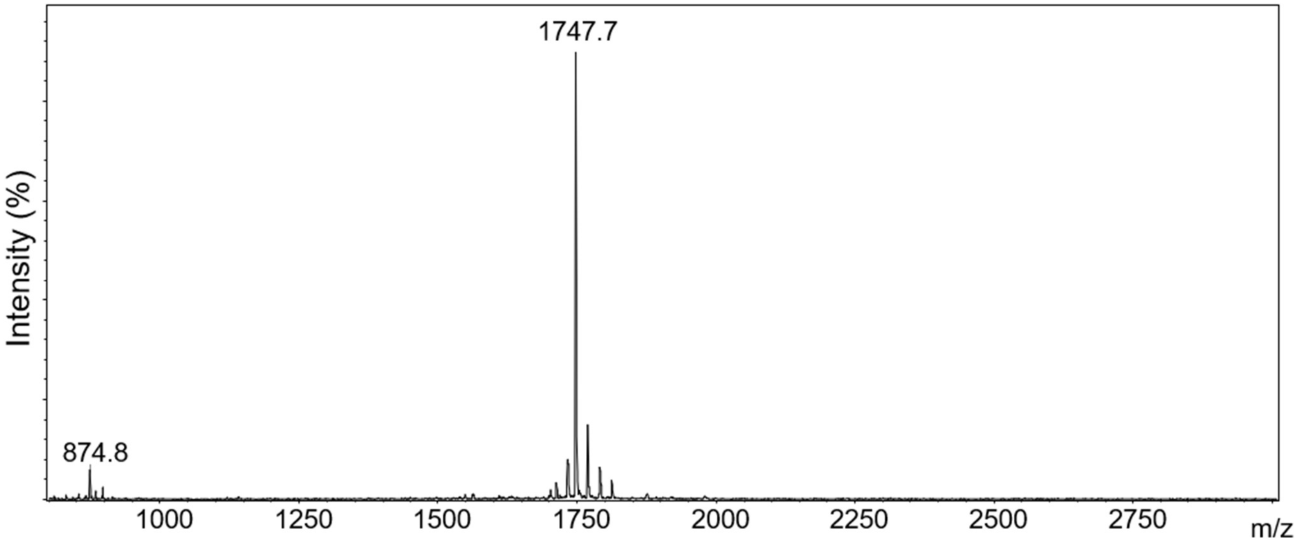

Figure S16. A) Analytical HPLC of the H4Abg16 peptide after RP-HPLC purification. **B)** MALDI-TOF MS spectra of the purified H4Abg16 peptide.

5. Time course plot of KAT8-catalysed acetylation

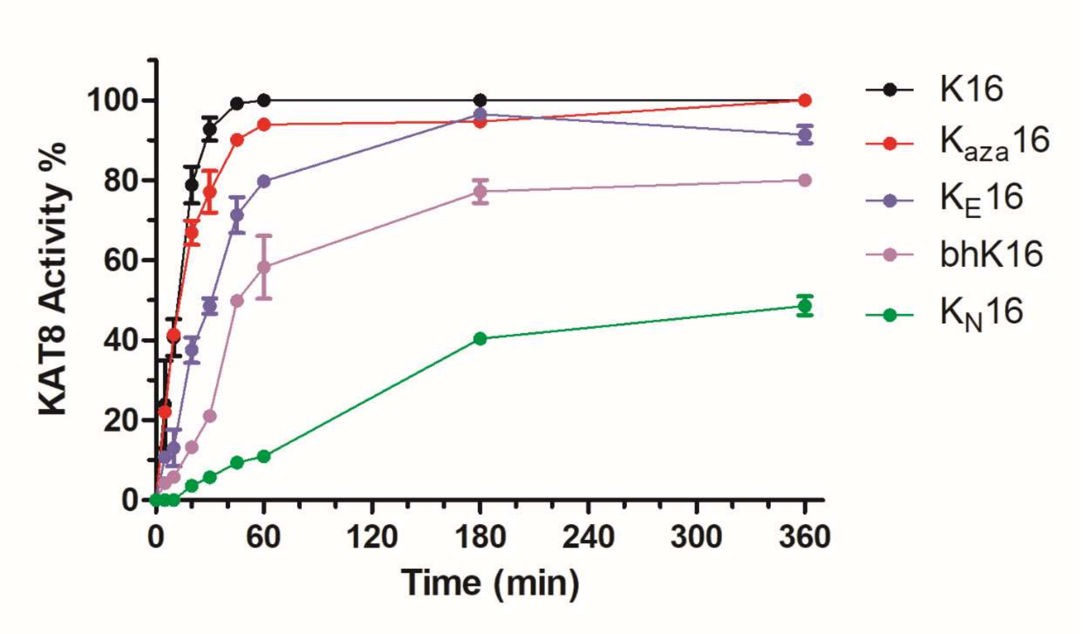

Figure S17. Time course analysis of KAT8-catalysed acetylation reactions of unnatural lysine analogues on the H4K16 sequence. The assay was performed in replicates under standard conditions (KAT8 2µM, histone peptide 100 µM. AcCoA 300 µM), and aliquotes of the reaction mixtures were quenched and analyzed by MALDI-TOF MS at set time points.

6. MALDI and crystallographic supporting figures

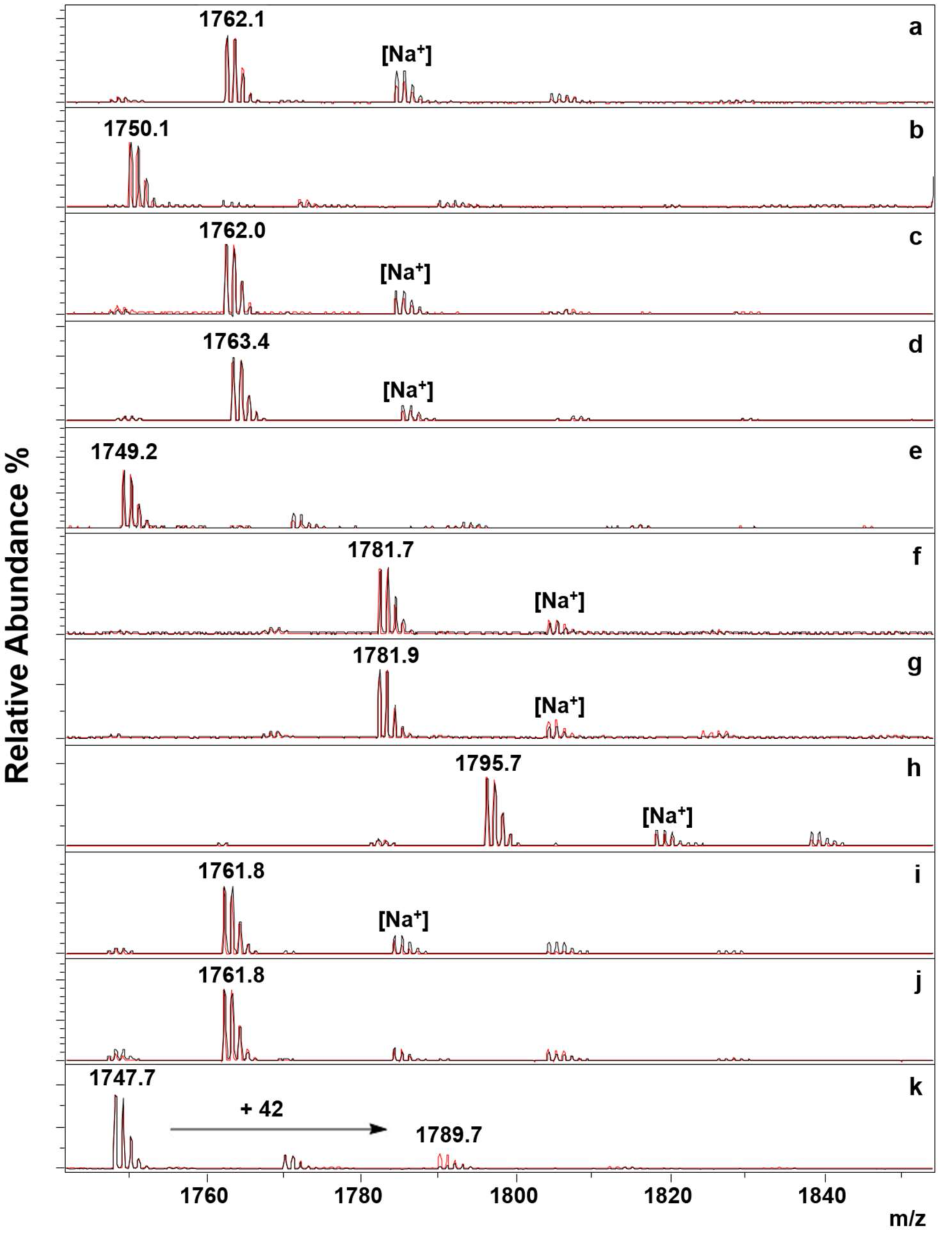

Figure S18. MALDI-TOF MS analysis of KAT8-catalysed reaction with higher concentration of KAT8 (10 µM) and extended time (6 hours). Overlaid spectra of reaction (red) and no enzyme control (black) of: a) H4Kme16; b) H4K_{oxy}16; c) H4hGln16; d) H4K_{COOH}16; e) H4K_{OH}16; f) H4F_{3a}16; g) H4F_{4a}16; h) H4$F_{3me\text{-}a}$16; i) H4K_{CMe}16; j) H4K_{NMe}16; k) H4Abg16.

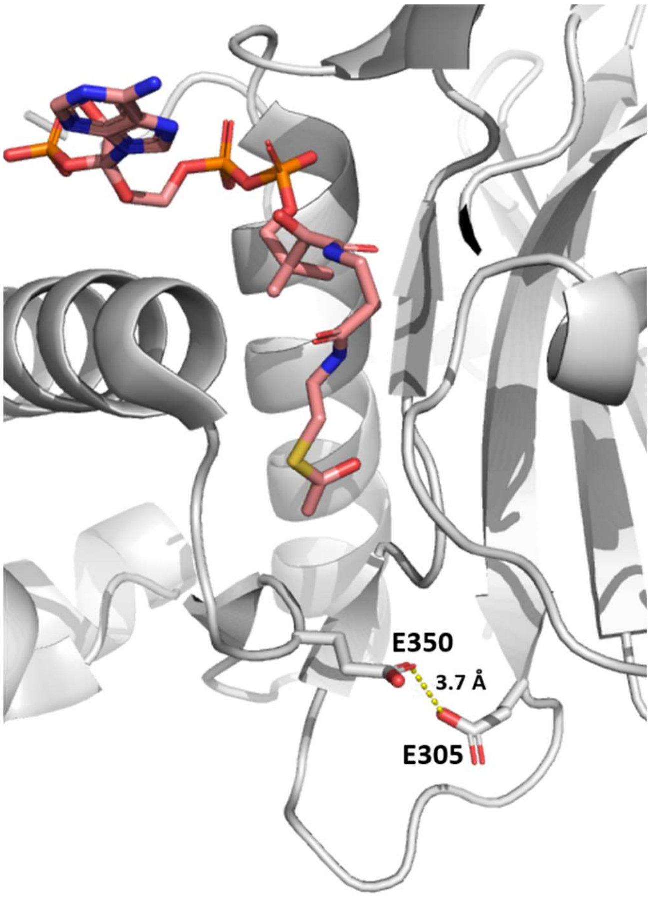

Figure S19. View on the crystal structure of the KAT8-AcCoA complex (KAT8: gray; AcCoA pink) (PDB: 2GIV), highlighting the two glutamic acid residues E350 and E305.

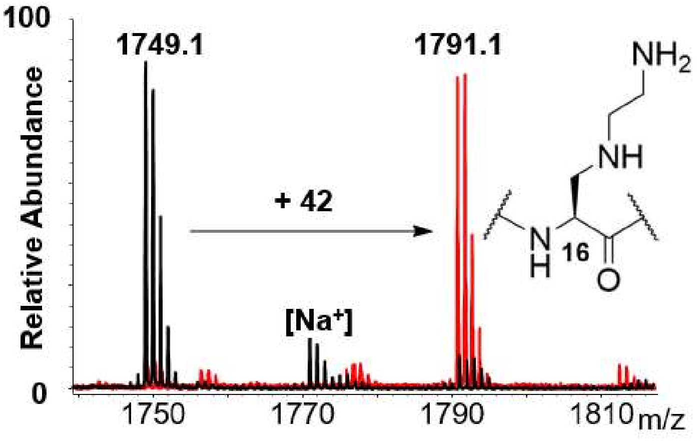

Fig S20. MALDI-TOF MS analysis of KAT8-catalysed quantitative reaction of H4K$_N$16 employing higher concentration of enzyme (10 μM) and extended time (6 hours). Overlaid spectra of reaction (red) and no enzyme control (back).

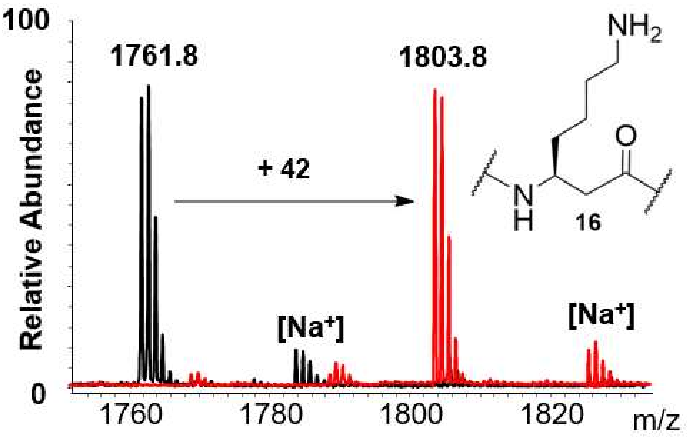

Figure S21. MALDI-TOF MS analysis of KAT8-catalysed quantitative reaction of H4βhK16 employing higher concentrations of enzyme (10 μM) and extended time (6 hours). Overlaid spectra of reaction (red) and no enzyme control (black).

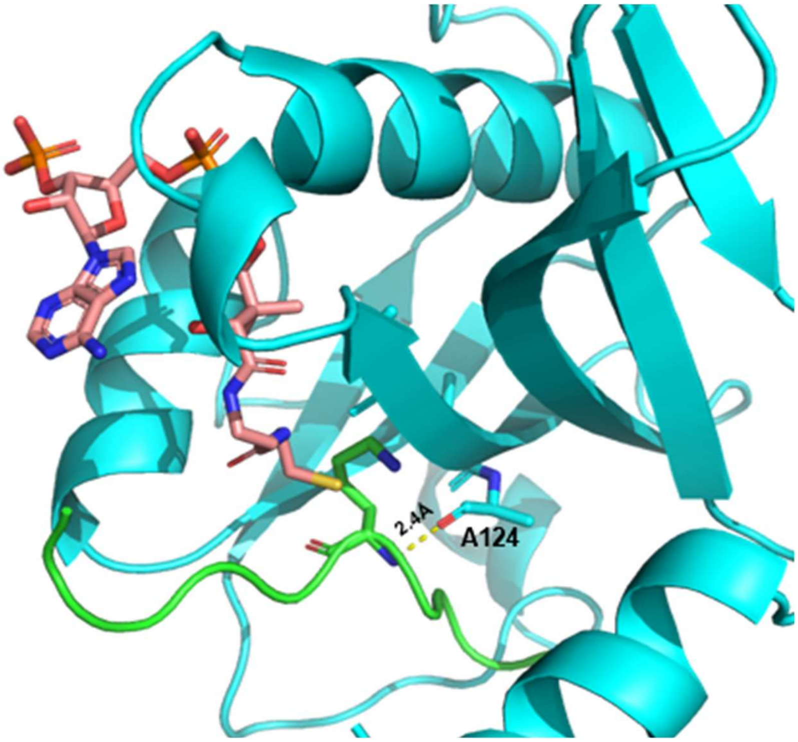

Figure S22. View on the crystal structure of the tKAT2A-H3K14-CoASH complex (tKAT2A: cyano; H3K14: green; AcCoA: pink) (PDB: 1QSN), highlighting the hydrogen bond interaction between the carbonyl group of the A124 residue and the alpha amino moiety of K14 on histone tail.

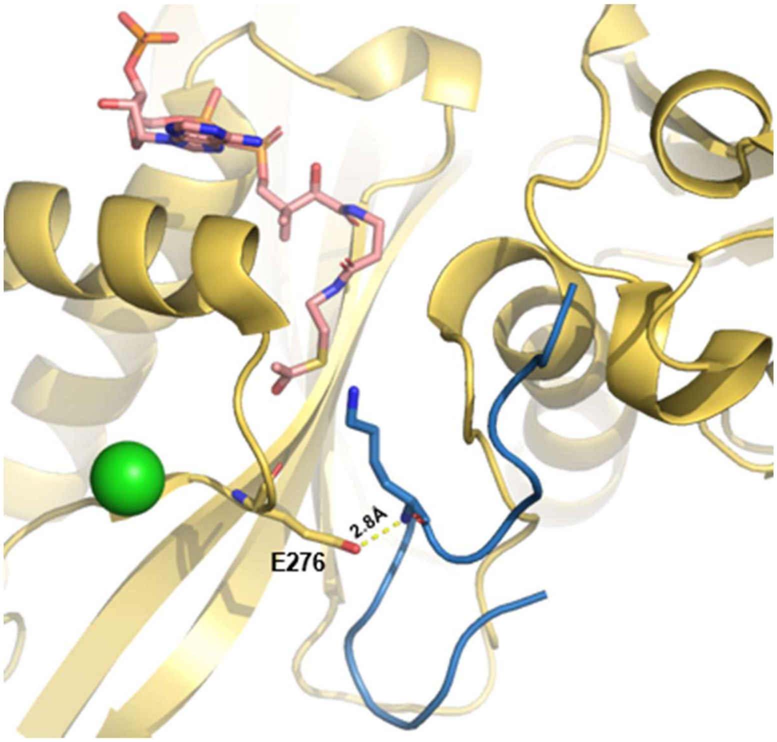

Figure S23. View on the crystal structure of the KAT1-H4K12-AcCoA complex (KAT1: yellow; H2K12: blue; AcCoA: pink) (PDB: 2P0W), highlighting the hydrogen bond interaction between the carbonyl group of the E276 residue and the alpha amino moiety of K12 on histone tail.

7. Inhibition plot

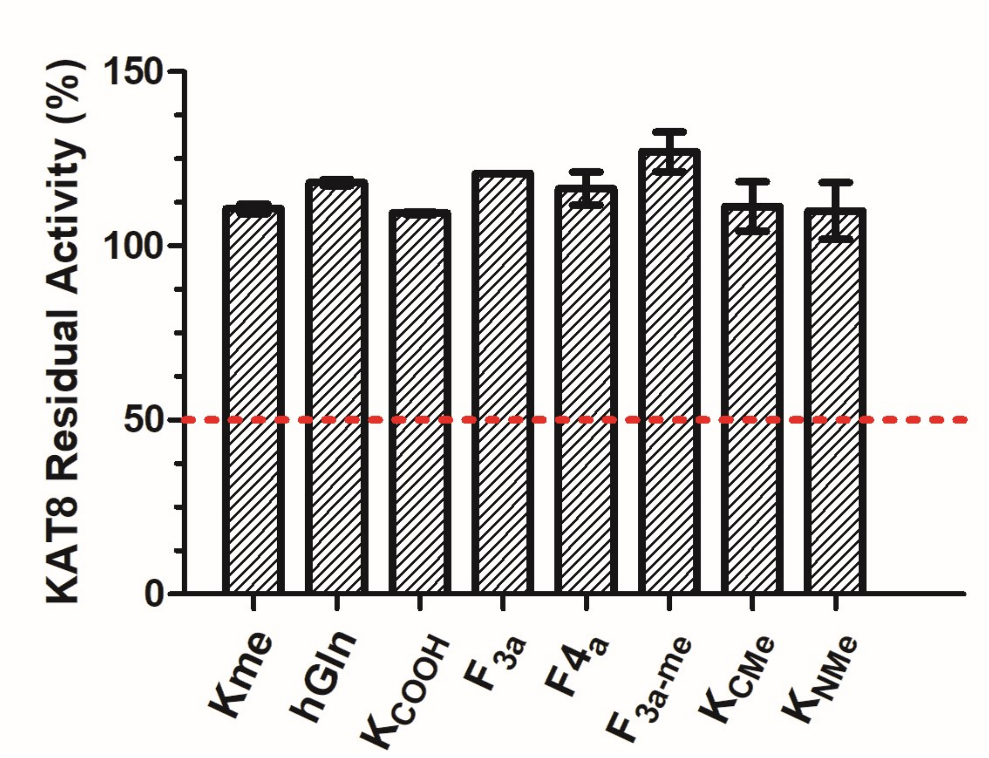

Figure S24. Enzyme residual activity upon incubation of KAT8 (0.2 μM) in the presence of AcCoA (300 μM), H4K16 (100 μM) and H4K*16 (100 μM) histone peptides. Reactions were quenched after 30 minutes.

8. NMR spectra

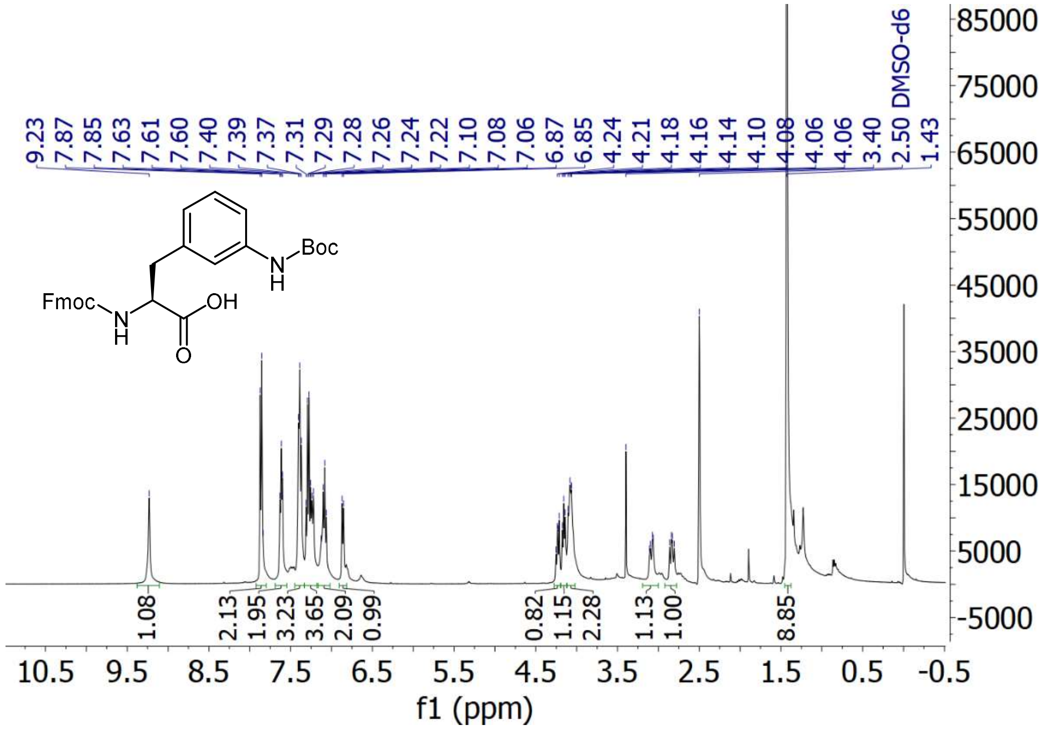

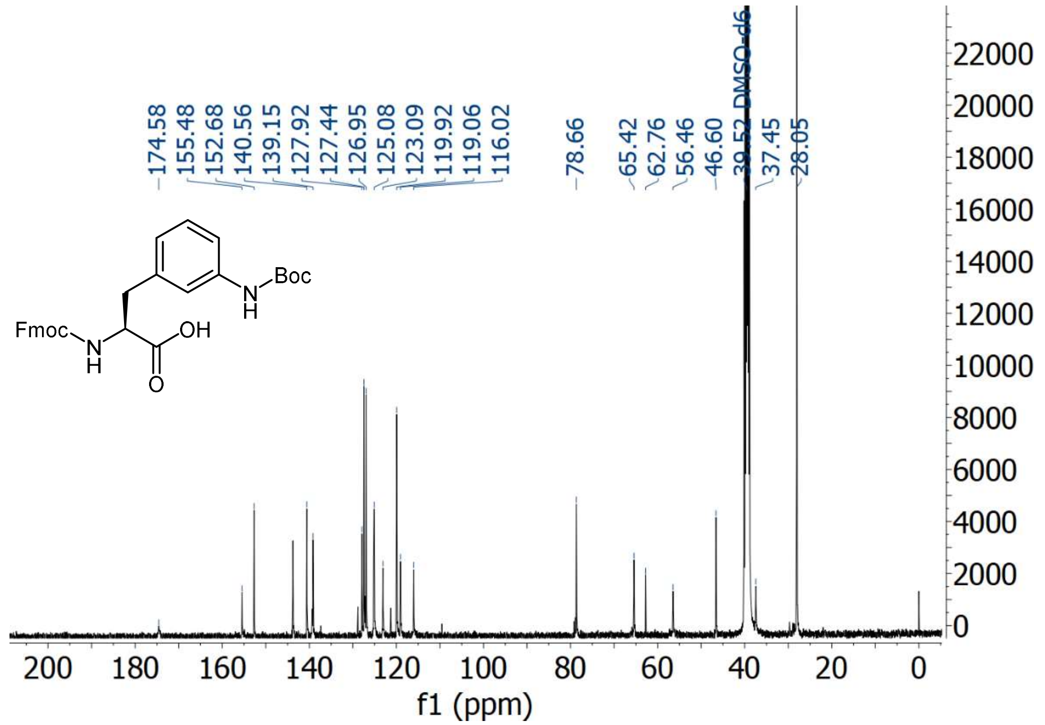

Supplement: Supplementary file 1 [file ijms-22-00846-s001.pdf]
